# Secretome from iPSC-derived MSCs exerts proangiogenic and immunosuppressive effects to alleviate radiation-induced vascular endothelial cell damage

**DOI:** 10.1186/s13287-024-03847-5

**Published:** 2024-07-29

**Authors:** Kshama Gupta, Ralph B. Perkerson, Tammee M. Parsons, Ramacharan Angom, Danilyn Amerna, Jeremy D. Burgess, Yingxue Ren, Pamela J. McLean, Debabrata Mukhopadhyay, Prasanna Vibhute, Zbigniew K. Wszolek, Abba C. Zubair, Alfredo Quiñones-Hinojosa, Takahisa Kanekiyo

**Affiliations:** 1 Department of Neuroscience, Mayo Clinic, 4500 San Pablo Road South, Jacksonville, FL 32224 USA; 2 Department of Cancer Biology, Mayo Clinic, 4500 San Pablo Road South, Jacksonville, FL 32224 USA; 3 Center of Regenerative Biotherapeutics, Mayo Clinic, 4500 San Pablo Road South, Jacksonville, FL 32224 USA; 4 Department of Quantitative Health Sciences, Mayo Clinic, 4500 San Pablo Road South, Jacksonville, FL 32224 USA; 5 Department of Radiology, Mayo Clinic, 4500 San Pablo Road South, Jacksonville, FL 32224 USA; 6 Department of Neurology, Mayo Clinic, 4500 San Pablo Road South, Jacksonville, FL 32224 USA; 7 Department of Neurosurgery, Mayo Clinic, 4500 San Pablo Road South, Jacksonville, FL 32224 USA

**Keywords:** iPSC-MSC, Radiation therapy, Angiogenesis, Inflammation, Secretome, Biotherapeutics

## Abstract

**Background:**

Radiation therapy is the standard of care for central nervous system tumours. Despite the success of radiation therapy in reducing tumour mass, irradiation (IR)-induced vasculopathies and neuroinflammation contribute to late-delayed complications, neurodegeneration, and premature ageing in long-term cancer survivors. Mesenchymal stromal cells (MSCs) are adult stem cells that facilitate tissue integrity, homeostasis, and repair. Here, we investigated the potential of the iPSC-derived MSC (iMSC) secretome in immunomodulation and vasculature repair in response to radiation injury utilizing human cell lines.

**Methods:**

We generated iPSC-derived iMSC lines and evaluated the potential of their conditioned media (iMSC CM) to treat IR-induced injuries in human monocytes (THP1) and brain vascular endothelial cells (hCMEC/D3). We further assessed factors in the iMSC secretome, their modulation, and the molecular pathways they elicit.

**Results:**

Increasing doses of IR disturbed endothelial tube and spheroid formation in hCMEC/D3. When IR-injured hCMEC/D3 (IR ≤ 5 Gy) were treated with iMSC CM, endothelial cell viability, adherence, spheroid compactness, and proangiogenic sprout formation were significantly ameliorated, and IR-induced ROS levels were reduced. iMSC CM augmented tube formation in cocultures of hCMEC/D3 and iMSCs*.* Consistently, iMSC CM facilitated angiogenesis in a zebrafish model in vivo. Furthermore, iMSC CM suppressed IR-induced NFκB activation, TNF-α release, and ROS production in THP1 cells. Additionally, iMSC CM diminished NF-kB activation in THP1 cells cocultured with irradiated hCMEC/D3, iMSCs, or HMC3 microglial lines. The cytokine array revealed that iMSC CM contains the proangiogenic and immunosuppressive factors MCP1/CCL2, IL6, IL8/CXCL8, ANG (Angiogenin), GROα/CXCL1, and RANTES/CCL5. Common promoter regulatory elements were enriched in TF-binding motifs such as androgen receptor (ANDR) and GATA2. hCMEC/D3 phosphokinome profiling revealed increased expression of pro-survival factors, the PI3K/AKT/mTOR modulator PRAS40 and β-catenin in response to CM. The transcriptome analysis revealed increased expression of GATA2 in iMSCs and the enrichment of pathways involved in RNA metabolism, translation, mitochondrial respiration, DNA damage repair, and neurodevelopment.

**Conclusions:**

The iMSC secretome is a comodulated composite of proangiogenic and immunosuppressive factors that has the potential to alleviate radiation-induced vascular endothelial cell damage and immune activation.

**Supplementary Information:**

The online version contains supplementary material available at 10.1186/s13287-024-03847-5.

## Introduction

Radiation therapy has been commonly used to treat all cancers in the central nervous system (CNS), including primary and metastatic brain tumours. The dosage of radiation therapy varies depending on the tissue type, stage, location, and size of the tumour, with a standard fractionated dosage of 2–5 Gy administered up to 60 Gy [1–5]. Despite the effectiveness of radiation therapy in eliminating tumours, its side effects have been well documented and include radiation-induced brain injury (RIBI) [6–10]. The pathophysiology of RIBI is similar to that of neurodegenerative disorders and involves extracellular matrix (ECM) alterations, blood‒brain barrier (BBB) damage, endothelial cell apoptosis, monocyte infiltration, neuroinflammation, metabolic dysfunction, senescence, and diminished neurogenicity [11–15] (Supplementary Fig. 1, Tables S1 and S2). Vasculopathies manifested by endothelial cell dysfunction, senescence, or cell death are major complications caused by radiation therapy [16–18] and can lead to chronic inflammation, brain parenchyma damage and cognitive decline at the late stage [19–25]. In contrast to high-dose irradiation (i.e., total dose of radiation therapy > 10 Gy) administered during cancer treatment regimens, which adversely impacts various brain regions, including the BBB and endothelial cells, low-dose irradiation (total dose of radiation therapy < 10 Gy, administered in fractions ≤ 0.1 to 2 Gy) is much less damaging [26]. Thus, efforts are underway to identify multimodal combinations of chemotherapy, immunotherapy and targeted therapy along with high-precision radiation to improve the efficacy of radiation therapy and reduce the overall dosage being administered [27–33]. Although several treatment approaches involving small molecules and high-precision radiation therapy, such as proton-minibeam therapy, are in preclinical development to overcome radiation-induced injuries, stem cell-based therapies have been proposed as new strategies to treat these pathogenic conditions and improve quality of life in postcancer care [25, 34–40].

Mesenchymal stromal cells (MSCs) are multipotent adult stem cells found in various tissues that originate from the mesodermal germ layer and can differentiate into connective tissues, skeletal muscle cells, and cells of the vascular system [41, 42]. MSCs support tissue integrity and homeostasis and accumulate during wound healing in response to stem cell mobilization and growth factors and in response to exercise [43, 44]. The therapeutic potential of bone marrow- and adipose tissue-derived MSCs is attributed to their ability to transdifferentiate, secrete trophic factors and induce immunosuppression [45–48]. Since infection, inflammation, and vascular derangement/degeneration are associated with radiochemotherapy-related tissue toxicity and posttreatment complexities, MSC-based therapies are being considered for cancer management [49–52]. However, controversies and challenges exist in the field. Several studies have shown that MSCs can exert both pro- and antitumorigenic effects and can be both pro- and anti-inflammatory, depending on their microenvironment, which indicates that the application of MSCs for cancer therapeutics should be considered with caution [53–56]. However, the ability of MSCs to migrate to irradiated tissues and to facilitate regeneration by differentiating into tissue-specific cells and by producing a supporting tissue architecture makes MSC-based therapies futuristic attenuators of radiotherapy-related late effects on cancer survivors [50, 57]. Challenges associated with obtaining effective biotherapeutic products from tissue-derived MSCs and complexities observed with cell-based therapies have shifted the focus towards cell-free therapy [58–63]. However, the MSC secretome can perform a multitude of functions, depending on the composition of its constituting analytes and the type of tissue insult for which it is administered. For instance, interleukin-6 (IL6), one of the main factors in the MSC secretome, can exert both pro- and anti-inflammatory effects in combination with other cytokines and soluble factors [64–66]. Thus, establishing a syngeneic and homogeneous source of MSCs that can be used to enhance the potential of the MSC secretome for achieving the desired therapeutic outcome is needed. The increasing success of induced pluripotent stem cell (iPSC) technology in augmenting cell-based therapies has indicated that iPSCs could serve as a homogenous, expandable, and genetically modifiable source of MSCs for cell therapy [67–70]. Since the MSC secretome contains tissue reparative factors, microparticles, and extracellular vesicles, we evaluated the potential of the iPSC-derived MSC (iMSC) secretome in immunomodulation and vasculature repair in response to radiation-induced alterations utilizing models of human monocytic cells and human brain endothelial cells. Insights from this study illuminate the possibility of harnessing iMSC-based regenerative therapies to combat radiation therapy-related organ damage and radiation therapy-induced progressive neurodegeneration.

## Materials and methods

### Generation of iPSC-derived MSCs (iMSCs)

Human iPSCs were generated from two healthy individuals (MC0039 and MC0063) and cultured in TeSR-E7 complete medium (STEMCELL Technologies, Vancouver, Canada) on Matrigel (Corning, Corning, NY, USA)-coated dishes, as reported previously [71]. The iPSCs were thawed in 2.5 ml of warm complete mTeSR1 supplemented with 10 µM Y27632 (1:1000), plated in 1 well of a Matrigel-coated 6-well plate, and incubated for 24 h. The next day, the media was changed to mTeSR1 without the ROCK inhibitor until confluency reached 80%. On Day 0 of iMSC generation, confluent cultures of iPSCs from at least 2 wells of a 6-well plate were detached by adding 1 ml/well Accutase solution (STEMCELL Technologies, 07920), the reaction was stopped using 1 ml of DMEM/F-12, and the cells were dispensed into single cells and centrifuged at 300 × g for 5 min. The cell pellets were resuspended in 1 ml of induction medium supplemented with 10 µM ROCK inhibitor, and the cell concentration was determined using an Invitrogen Countess II Automated Cell Counter (Thermo Fisher Scientific; AMQAX1000). Cells were distributed (100 µL per well) in 96-well round-bottomed culture plates at a concentration of 150,000 cells/mL of induction medium supplemented with 10 µM ROCK inhibitor to generate embryoid bodies (EBs). The plates were centrifuged at 100 × g for 5 min and incubated in a humidified incubator with 5% CO_2_ at 37 °C for 48 h. On Day 2, 4–5 EBs were transferred to non-tissue-treated 6-well plates and incubated for an additional 3 days in induction medium. On Day 5, EBs from each well were collected in a 1.5 ml flip-top tube, slightly disrupted by pipetting with a 10 µl pipet tip, dispensed in 1 ml of induction medium, and plated in a 1% gelatine-coated 6-well plate. On Day 8, the spent media with unattached EBs were removed from the wells, and freshly prepared differentiation medium (reprogramming medium) for iMSCs was gently added to each well without disturbing the adhered EBs. The EBs were cultured at 37 °C in a humidified incubator with 5% CO_2_ for 3 days. On Day 11, the media were replaced with regular MSC maintenance media, and the cells were cultured until 90% confluence with intermittent changes of media (twice per week). Once confluent, the centrally located EBs were detached using a pipet tip and washed away, leaving the peripheral MSC-like cells, which were then detached with 500 µL of TrypLE, pelleted by centrifugation at 300 × g for 5 min, resuspended in MSC media, and plated in T25 flasks. The iMSCs were subsequently passaged twice in a T75 flask to obtain a stable iMSC line. After 3 passages, the iMSCs were analysed for MSC surface markers using a standard FACS procedure. FACS was performed utilizing a Human MSC Analysis Kit (BD Stemflow™, 562,245) according to the manufacturer’s protocol, and data were acquired with an Attune NxT Flow Cytometer to confirm > 90% positivity for the markers CD73, CD90, and CD105 and < 5% positivity for the markers CD34, CD45, CD11b, CD19, and HLA-DR. iMSC lines that passed the assessment for surface marker expression were stored frozen in CryoStor® CS5 cell cryopreservation media at a concentration of 1 × 10^6^ iMSCs/mL. The trilineage differentiation potential of the iMSCs was further confirmed via Oil Red O staining for adipogenesis, Alizarin Red S staining for osteogenesis, and Alcian blue staining for chondrogenesis using StemPro® Differentiation Kits (Adipogenesis, Gibco A1007001; Chondrogenesis, Gibco A1007101; Osteogenesis, Gibco A1007101) prior to functional characterization. Detailed information is provided in Supplementary Fig. 2 and Table S3.

### Cell culture

The human brain endothelial cell line hCMEC/D3 was purchased from Millipore Sigma (SCC066, Sigma‒Aldrich) and expanded in complete endothelial growth media (CC-3124, Lonza) according to standard cell culture practices. Human THP1 NF-κB-Luc2 cells and the human microglial cell line HMC3 were purchased from ATCC (Rockville, MD) and grown according to the ATCC protocol in complete RPMI media or complete DMEM-F12, respectively. Adipose tissue-derived MSCs (AD-MSCs) and bone marrow-derived MSCs (BM-MSCs) were obtained from the Human Cell Therapy Lab (HCTL) at the Mayo Clinic, Jacksonville. MSCs, irrespective of their source and origin, were maintained in α-MEM supplemented with 5% FBS. All multiwell cell cultures were conducted in 96-well flat-bottom plates with a Nunclon delta surface (167,008, Thermo Fisher Scientific, Waltham, MA, USA) in their respective culture media (Table S4). Spheroid 3D cultures of hCMEC/D3 were established by modifying the endothelial barrier 3D-spheroid modelling protocol described previously [72]. hCMEC/D3 cells were seeded at optimized densities between 20,000 and 30,000 cells/well in U-bottom, ultralow attachment 96-well plates (Corning, Costar 7007). The cells were cultured for 24 h in complete endothelial growth media or in conditioned media and basal RPMI. After 24 h, spheroids were stained using the cell permeant stain calcein AM (Invitrogen, 65-0853-39) or a live/dead cell imaging kit with calcein AM as a live cell indicator and BOBO-3 iodide as a dead cell indicator (Invitrogen, R37601) to evaluate cell viability and spheroid integrity (spheroid cell‒cell adherence).

### Conditioned media preparation

For the preparation of conditioned media, MSCs from the respective sources were cultured in maintenance media to 80% confluency in a T75 standard cell culture-treated flask. The cells were gently washed with phosphate-buffered saline, the media were changed to 10 ml of RPMI media (without FBS or any additive supplements), and the cells were cultured in a humidified cell culture incubator with 5% CO_2_ for 5 days. On Day 5, the conditioned media (CM) was harvested from the flask, collected in a 15 ml Falcon tube, and cleared by centrifugation at 1000 × g for 10 min in a refrigerated centrifuge with a hanging bucket rotor at 4 °C. The filtered (spin-cleared) CM was then aliquoted in prechilled 1.5 ml microfuge tubes and stored at −80 °C. For intermittent collection, aliquots of CM were harvested at 24 h and 48 h from the same culture flask, spin cleared and stored. After the conditioned media were harvested, the cells were stained with calcein AM viability dye (Invitrogen, 65-0853-39) and imaged for cell morphology using EVOS FL (at 4 × or 10 × magnification) and a Keyence microscope (at 2 × or 20 × magnification). The cells were also subjected to FACS analysis to confirm the expression of MSC cell surface markers.

### X-ray irradiation, endothelial cell viability and adherence

X-ray irradiation (IR) was applied to endothelial cells cultured for 24 h as monolayers or spheroids utilizing an X-RAD 160 X-ray Biological Irradiator (Precision X-ray Inc., North Branford, CT, USA) at a maximum mA = 18.7, maximum kV = 160, and dose rate of 403.8 cGy/min. After IR, the cells were washed with PBS, treated with conditioned media or RPMI media (basal media control) and continually cultured for 24 h post-IR treatment. Spheroid cell viability (CellTiter-Glo®, G7572, Promega), caspase 3/7 activity (Caspase-Glo® 3/7 Assay System, G8090, Promega), and ROS production (ROS-Glo™ H2O2 Assay, G8820, Promega) were assessed according to the manufacturer’s protocols to evaluate the effect of the CM treatment on the growth of endothelial cells. hCMEC/D3 cultured as spheroids were subjected to live/dead staining (Invitrogen, R37601) as described in Methods section “Cell culture”). IR of all other cell types, including THP1 cells and iMSCs, was performed similarly using Precision X-RAD 160 at the doses optimized and specified in each experiment.

### Angiogenesis assessment in vitro and in vivo

#### Tube formation assay

The endothelial cell tube formation assay was adapted from published protocols [73]. Briefly, a reduced growth factor basement membrane, Cultrex RGF BME-type2 (R&D Systems, 3533-005-02), was thawed overnight at 4 °C. Flat-bottom 96-well plates were chilled, and the cold BME was layered at a volume of 40 µl per well 2 h prior to the start of the assay. The basement membrane coating was allowed to solidify for 1 h by placing the plate at 37 °C in a humidified incubator with 5% CO_2_. hCMEC/D3 cells were cultured in complete EGM2 media to 80% confluence. One hour prior to the assay, the cells were starved by the addition of serum-free basal EGM2 media. Cells were harvested using trypsin EDTA (25,200-056, Gibco) and washed with PBS, and the cell count was estimated using a *Vi*-*CELL BLU* cell *counter* (Beckman Coulter, C19196). For visualization, calcein AM was added to the basal media at a final concentration of 2 µg/mL, and the cells were stained at 37 °C with 5% CO_2_ for 1 h prior to the start of the assay. For coculture with iMSCs, iMSCs were starved in serum-free basal α-MEM supplemented with 5 µM *CellTracker Red* CMTPX (Invitrogen, C34552) and stained for 1 h at 37 °C in a humidified incubator containing 5% CO_2_. The harvested and counted hCMEC/D3 cells were treated with IR at a dose of 5 Gy or not irradiated (0 Gy) as a control. Aliquots of stored frozen iMSC-derived CM were thawed and brought to room temperature. hCMEC/D3 cells were stained with calcein AM and cocultured with iMSCs stained with CellTracker Red at a ratio of 2:1 (20,000 hCMEC/D3:10,000 iMSCs), followed by supplementation with iMSC-conditioned media or RPMI control media. Tube formation began within 2–4 h and depended on the angiogenic factor concentration in the conditioned media, in which peak tube formation occurred between 12 and 24 h and was withdrawn from 36 to 48 h. Images were acquired at 6 h for the early time point and at 24 h and 36 h for the late time points of tube formation using a Keyence microscope.

#### Zebrafish model

A *Tg*(*flk1:EGFP*) transgenic line of zebra fish, *Danio Rerio*, expressing eukaryotic green fluorescent protein (EGFP) under the control of the *Flk1/vegfr2* gene promoter was maintained at 28 °C on a 14/10-h (light/dark) cycle in zebrafish water (Zf-H_2_O) according to a standard protocol [74–76]. Zebrafish were crossed by natural mating. At 24 h postfertilization (hpf), the embryos were collected and dechorionated using pronase enzyme degradation. The embryos were then suspended at a density of 25 embryos per 2.5 ml of RPMI media or iMSC CM in a 12-well plate; zebrafish water (Zf-H_2_O) was used as a positive control for embryo growth. The sample size of *n* = 25 was decided per treatment group to begin with, as considered optimal for such studies. Any developmentally abnormal embryos were not included in the study. *n* ≥ 3 embryos per treatment group were imaged and, quantified, and figure represents data from two independent experiments. For imaging, the zebrafish embryos were anesthetized by using pharmaceutical grade Tricaine/Finquel MS222, 0.015%. EGFP expression was evaluated after 48 hpf utilizing a confocal microscope (LSM 880, Zeiss). EGFP intensity in the head and trunk regions was analysed using ImageJ. At the endpoint, the zebrafish larvae were euthanized by immersing in sodium hypochlorite solution(bleach) for 5 min as the institutional ethical committee approved. The work has been reported in line with the ARRIVE guidelines 2.0.

### Immunomodulation assay

The human monocytic cell line THP1 (ATCC, TIB-202) and its derivative reporter line THP1 NF-κB-Luc2 (ATCC, TIB-202-NF-kB-LUC2™) were utilized to assess the immunomodulatory effects of iMSC CM.

#### Effect of iMSC CM on IR-induced NF-κB activation in THP1 cells

THP1 NF-κB-Luc2 reporter cells cultured in complete RPMI media were irradiated utilizing an X-RAD 160 X-ray Biological Irradiator (Precision X-ray Inc., North Branford, CT, USA) at various doses (0 Gy, 2.5 Gy, 5 Gy, 10 Gy, and 15 Gy), as indicated in the respective experiments. The cells were then washed, counted, suspended in iMSC CM or RPMI media alone, and seeded on a 96-well plate at a concentration of 50,000 cells/well. After 6 h, the reporter activity was measured using a One-Glo luciferase assay system (Promega, E6120) according to the manufacturer’s protocol. For the time-point experiment, cells were harvested intermittently at 2 h, 4 h, 6 h or 18 h, and reporter activity was measured.

#### Effect of iMSC CM on IR-induced NF-κB activation in THP1 cells cocultured with iMSCs, hCMEC/D3, and/or HMC3 cells

hCMEC/D3 and iMSCs were seeded for monoculture or coculture (at a ratio of 1:1) at a concentration of 50,000 cells/well in 96-well plates. After 24 h, the two plates were irradiated at 0 Gy or 5 Gy utilizing an X-RAD 160 X-ray Biological Irradiator. The cells were washed with PBS and then overlaid with THP1 NF-κB-Luc2 reporter cells resuspended in iMSC CM or RPMI media. After 24 h, the reporter activity was measured using a One-Glo luciferase assay system. HMC3 human microglia were cultured at a concentration of 20,000 cells/well in 96-well plates and allowed to grow to 90% confluence. The cells were then irradiated at 0 Gy or 5 Gy and overlaid with THP1 NF-κB-Luc2 reporter cells resuspended in iMSC CM or RPMI media. In the cell control wells, no THP1 NF-κB-Luc2 cells were overlaid on HMC3 cells, and the cells were allowed to grow in the presence of IR alone. After 48 h, reporter activity was measured in wells with overlaidTHP1 reporter cells. In the cell control wells, HMC3 cell viability was measured using CellTiter-Glo (Promega, G7572), and the estimated reporter activity was normalized to the HMC3 cell viability.

#### Effect of iMSC CM on IR-induced THP1 cell activation

(i) For the cytokine release assay, THP1 cells cultured in complete RPMI media were subjected to 1 h of serum starvation. The cells were then irradiated at 0 Gy or 15 Gy, centrifuged, counted, resuspended in iMSC CM or RPMI media, and seeded at a concentration of 50,000 cells/well in a 96-well plate. After 24 h, the culture plate was centrifuged at 1000 × g for 5 min, and the culture medium from each well was harvested. The TNF-α levels in the culture medium were measured using a Lumit™ TNF-α (human) immunoassay kit (Promega, W6050). The viability of cells in each well was measured using CellTiter-Glo (Promega, G7572). The data are presented as TNF-α levels normalized to the cell viability in each well. (ii) For the cell clustering assay, THP1 cells cultured in complete media were serum starved in RPMI media supplemented with 5 µM *CellTracker Red* CMTPX (Invitrogen, C34552) for 1 h. The cells were then harvested, washed, and resuspended in basal RPMI media followed by stimulation with lipopolysaccharide (LPS:005:B5, Sigma Aldrich, L2880) at various concentrations (0 µg/ml, 5 µg/ml, 10 µg/ml, 20 µg/ml and 40 µg/ml). After 24 h, cell clustering was observed using EVOS FL (4x). THP1 cells were starved in serum-free medium, resuspended in iMSC CM or RPMI media, and stimulated with LPS to evaluate the effect of iMSC CM on cell clustering. After 24 h, images were acquired using an EVOS FL system (4x). After stimulation with LPS (0 µg/ml or 5 µg/ml), the number and size of the THP1 cell clusters were measured using ImageJ.

### Characterization of the iMSC secretome

Factors secreted by MSCs into the CM were evaluated utilizing a targeted approach. The harvested and filtered CM was subjected to cytokine array profiling for the initial screening and identification of secretome factors using an 80-target spotted membrane-based human cytokine antibody array (ab133998, Abcam). Subsequently, angiogenesis and immunomodulatory signatures were assessed using a 42-target spotted membrane-based human cytokine antibody array (ab133997, Abcam). All control and test dot blots were performed using this cytokine array according to the manufacturer’s protocol. The membranes were developed using chemiluminescence, and images were acquired using a ChemiDoc™ MP imaging system (Bio-Rad). The identified positive signals were quantified using ImageJ.

#### In silico analysis of soluble proteins identified in the iMSC secretome

Protein–protein interactions were evaluated, and GO-biological process and Reactome pathways were identified utilizing the STRING (https://string-db.org/) database to evaluate interactions between factors secreted in iMSC CM. Pathway engagement was further assessed using Pathway Commons (https://www.pathwaycommons.org/).

#### In silico analysis of regulatory elements in the iMSC secretome

Promoter regions (−5000 to + 1000) common to 6 secretome factors (MCP1, IL6, IL8, ANG, GRO-alpha, and RANTES) were obtained using ExPASy to explore the regulatory elements in the gene promoters of analytes identified in the iMSC secretome. The top-ranked transcription factor (TF) binding motifs in the promoter region were identified using tools available in the MEME suite 5.5.4 based on the following analysis methods: SEA, simple enrichment analysis; AME, analysis of motif enrichment; XTREME, motif discovery and enrichment analysis; and GLAM2, gapped local alignment motifs. The motif databases used were JASPAR CORE 2022 and Human, HOCOMOCO v11. Common TF-binding sites in the 3–4 kb promoter region of the same prime 6 secretome factors were identified by Swiss Regulon to further verify the presence of regulatory elements. The gene transcription regulation database (GTRD) was utilized to identify the frequency of TF-binding sites for the top-ranked (common) transcription factor motifs identified utilizing the MEME suite 5.5.4.

#### Comodulation of soluble factors present in the iMSC secretome

An inhibitor of the top-ranked transcriptional regulator, apalutamide (T2339, TargetMol), was diluted in DMSO, mixed in RPMI medium and administered to the iMSC cells at concentrations of 0, 5 and 25 µM, as indicated, to validate the roles of regulatory elements in the gene promotes of analytes identified in the iMSC secretome. Conditioned media were harvested from iMSCs treated with or without apalutamide on Day 5 and subjected to analysis of secretome factors using a 42-target spotted membrane-based human cytokine antibody array (ab133997, Abcam). An assessment of whether individual secreted factors, such as IL6, can be responsible for the proangiogenic phenotype exhibited by iMSC CM or whether the functional outcome of the iMSC secretome is a composite effect of various comodulated factors present was performed by conducting IL6 neutralization and pull-down assays after mixing a human IL6 antibody (R&D systems, MAB2061) in conditioned media overnight at 4 °C and immunoprecipitating IL6-antibody complexes with Protein G beads. Clearance of IL6 from the conditioned media was validated using cytokine array-based dot blotting. The effect of IL6-depleted conditioned media versus neat (complete) conditioned media on endothelial cell viability was evaluated using CellTiter-Glo® (G7572, Promega).

### RNA sequencing

RNA-Seq was performed for three technical replicates of AD-MSCs, M-MSCs, and the iMSC line (MC0039). RNA was isolated using a Direct-zol™ RNA Miniprep kit (Zymo Research, R2053). The samples were processed according to the manufacturer’s protocol, diluted appropriately, and submitted to the Sequencing Core Facility at the Mayo Clinic Rochester.

#### RNA sequencing, quality control and normalization

mRNA samples were sequenced using an Illumina HiSeq 4000 platform. Reads were mapped to the human reference genome hg38. Raw gene read counts, along with sequencing quality control, were generated using the Mayo Clinic RNA-Seq analytic pipeline MAP-RSeq Version 3.0 [77]. Conditional quantile normalization (CQN) was performed on the raw gene counts to correct for gene length differences, GC bias, and global technical variations and to obtain similar quantile-by-quantile distributions of gene expression levels across samples [78]. Based on the bimodal distribution of the CQN-normalized and log2-transformed reads per kilobase per million (RPKM) gene expression values, genes with an average log2 RPKM ≥ 2 in at least one group were considered expressed. Using this selection threshold, 16,603 genes were included in the downstream analysis.

#### Differentially expressed gene and pathway analyses

Analyses of differentially expressed genes were performed using Partek Genomics Suite (Partek Inc., St. Louis, MO). Gene expression between genotypes was calculated using analysis of variance (ANOVA) models. Pathway analyses of differentially expressed genes were performed via Ingenuity Pathway Analysis (IPA) and validated with the Search Tool for the Retrieval of Interacting Genes/Proteins (STRING) database to identify GO biological processes and Reactome pathways.

#### Data annotation and representation

Based on the adjusted *P* values, RNA-Seq data were sorted to identify genes whose expression did not change between AD-MSCs and BM-MSCs. From this gene list, the data were sequentially sorted to obtain genes that were significantly upregulated or downregulated in the tissue-derived MSCs compared to the iMSCs. Functional annotation was performed for the top 2000 genes most significantly upregulated and downregulated in tissue-derived MSCs versus iMSCs using the STRING database, and the top 30 biological processes (GO) and Reactome pathways upregulated or downregulated in tissue-derived MSCs relative to iMSCs are represented in bar graphs. (i) Volcano plots were constructed to present the negative log_10_ adjusted P value against the log fold change of normalized read counts obtained for all genes with differentially regulated expression between AD-MSCs and iMSCs and between BM-MSCs and iMSCs. (ii) Differential expression of transcription factors in iMSCs was plotted in bar graphs showing the negative log_10_ adjusted *P* value and fold change in normalized read counts for the expression of transcription factors of the TFAP2 and GATA families for comparisons of AD-MSCs and BM-MSCs versus iMSCs. The negative log_10_ adjusted *P* values against the log fold change of the normalized read counts obtained for genes related to the zinc finger nuclease (ZNF) and homeobox (HOX) families of transcription factors are presented in volcano plots for the comparisons of AD-MSCs versus the iMSCs and BM-MSCs versus the iMSCs. (iii) The differential expression of secretome factors in iMSCs is plotted in bar graphs showing the negative log_10_ adjusted *P* value and fold change for the expression of iMSC secretome analytes between the AD-MSCs and BM-MSCs versus iMSCs.

### Endothelial cell kinome

hCMEC/D3 cells were cultured in 6-well plates in complete endothelial growth medium until they reached 80% confluence. The cells were washed with PBS, and the media were replaced with iMSC-CM or RPMI basal media. After 24 h, the cells were washed with PBS supplemented with phosphatase inhibitors, detached in 1 ml of cold PBS using a cell scraper, dispensed in a 1.5 ml microfuge tube and pelleted at 500 × g for 5 min. The cell pellets were lysed in 200 µl of RIPA buffer (Abcam, ab156034) supplemented with a phosphatase inhibitor cocktail (Thermo Fisher Scientific, 1,861,281) and incubated on ice for 10 min. The lysed cells were then centrifuged at 10,000 rpm at 4 °C to remove any unlysed cell debris and transferred to a fresh microfuge tube. The phosphorylation profiles of kinases and their substrates in the lysates were assessed using the Proteome Profiler Array provided in the Human Phospho-kinase array kit (R&D Systems, ARY003C). Prior to performing the kinase array, the protein concentration was measured using a Pierce™ BCA protein assay (Thermo Fisher Scientific, 23,227). The protein lysate was then diluted in water to obtain a 1 ml suspension containing 200 µg/ml total protein, which was subsequently added to nitrocellulose membranes spotted in duplicate with antibodies against 37 kinase phosphorylation sites and 2 related total proteins, as provided by the kit. The procedure was performed according to the manufacturer’s protocol. The membranes were developed using chemiluminescence, and images were acquired using a ChemiDoc™ MP imaging system (Bio-Rad). At least two independent sets of lysates were prepared with and without treatment with iMSC CM. The identified positive signals were quantified using ImageJ, and the average signal from two independent experiments was assessed for the kinome profile.

### Statistical analysis

Data were analysed using GraphPad Prism, and one-way or two-way ANOVA was utilized to compare group means; Student’s t test was used where appropriate. Statistical significance is indicated as *p < 0.05, **p < 0.01, ***p < 0.001, and ****p < 0.0001. Significant differences (asterisks) between irradiated and nonirradiated (0 Gy) conditions within each group are represented as a colour code (blue for RPMI media and red for iMSC CM) where needed. All experiments were performed at least two or more times independently to identify trends in the observed data, and the data are presented as the means of at least two independent experiments with error bars indicating the standard errors of the means.

## Results

### IR causes brain endothelial cell damage

**Fig. 1:**
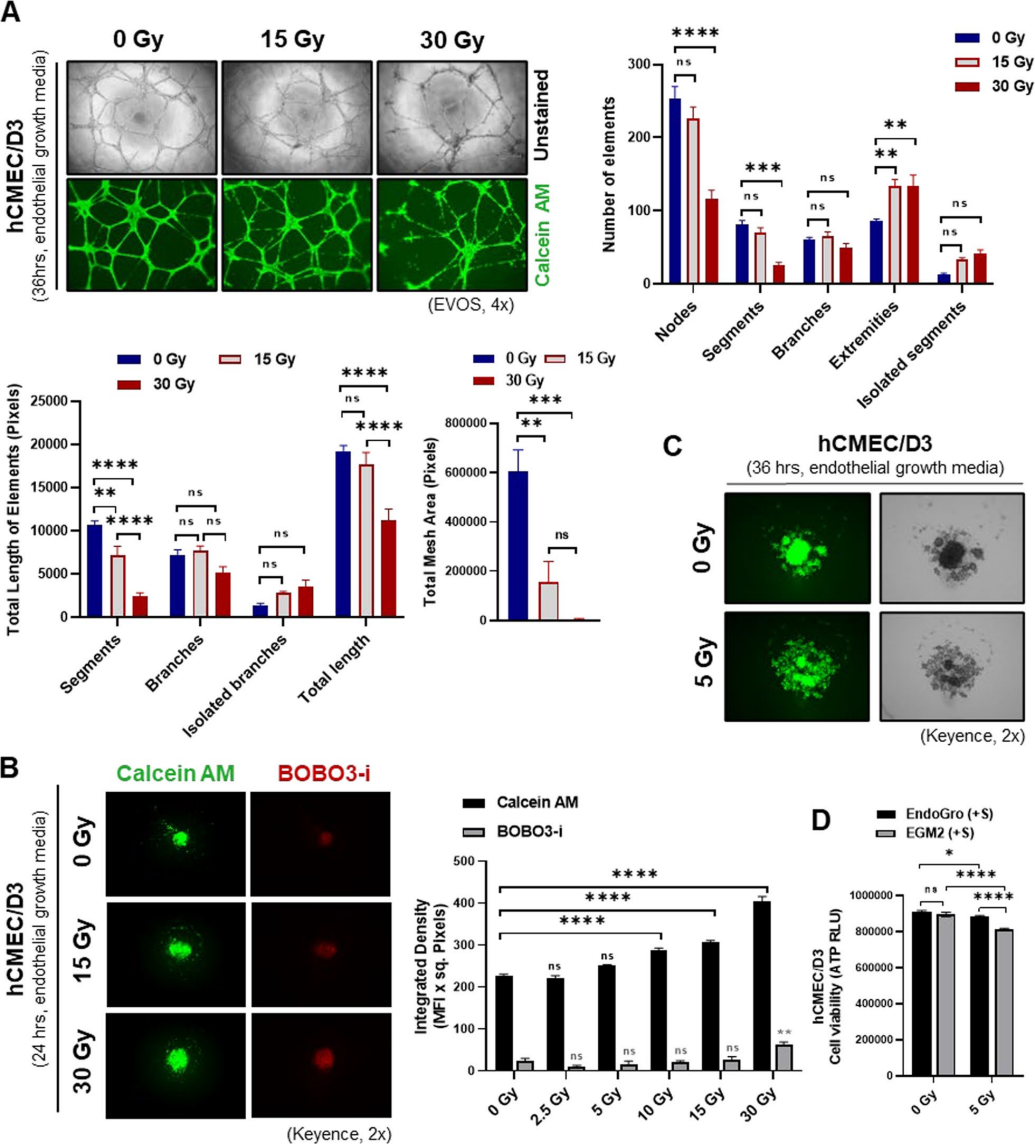
Vascular endothelial cell phenotype in response to irradiation: **A**, Endothelial cell tube formation by hCMEC/D3 cells was assessed 24 h after IR at 15 and 30 Gy, and the numbers of nodes and segments, lengths of segments and total mesh area, and number of extremities were calculated. **B**, **C**, Compact spheroid formation and spheroid disruption in hCMEC/D3 cells were assessed by staining with calcein AM and BOBO3-i 24 h after IR at 10, 15, and 30 Gy (**B**) and 36 h after IR at 5 Gy (**C**). **D**, hCMEC/D3 viability was assessed by measuring ATP production 24 h after IR at 5 Gy in EndoGro or EGM2 medium**.** The data are presented as the means ± SEMs (*n* = 3/group); *p < 0.05, **p < 0.01, ***p < 0.001, and ****p < 0.0001 according to one-way ANOVA or two-way ANOVA with Tukey’s test

A tube formation assay was performed in complete endothelial growth medium utilizing the human brain endothelial cell line hCMEC/D3 to examine the effect of IR on the vasculature in vitro. Compared to those in the control nonirradiated group (0 Gy), a significant decrease in angiogenic tube formation was observed 36 h after the administration of 15 Gy or 30 Gy of single-dose IR, with significant decreases in the numbers of nodes and segments, the length of segments and the total mesh area and an increase in the number of extremities (Fig. 1A, Supplementary Fig. 3A). Since angiogenic tube formation requires effective cell‒cell and cell–basement membrane interactions, we evaluated the effect of IR on hCMEC/D3 adherence utilizing a spheroid formation assay. Increasing doses of IR led to reduced spheroid compactness and enhanced spheroid disruption, as indicated by the increased area coverage of cells stained with calcein AM, and a loss of cell integrity, as indicated by increased BOBO3-iodide staining. hCMEC/D3 spheroids were significantly altered 24 h after IR administration at doses ≥ 10 Gy, and spheroid disruption was evident with IR at 5 Gy when monitored for 36 h (Fig. 1B, C and Supplementary Fig. 3B). When hCMECs/D3 were irradiated at 0 Gy or 5 Gy, a decrease in viability was observed irrespective of the culture medium (EndoGro or EGM2) 24 h after IR at 5 Gy (Fig. 1D).

### iMSC secretome treatment attenuates IR-induced brain endothelial cell damage

**Fig. 2:**
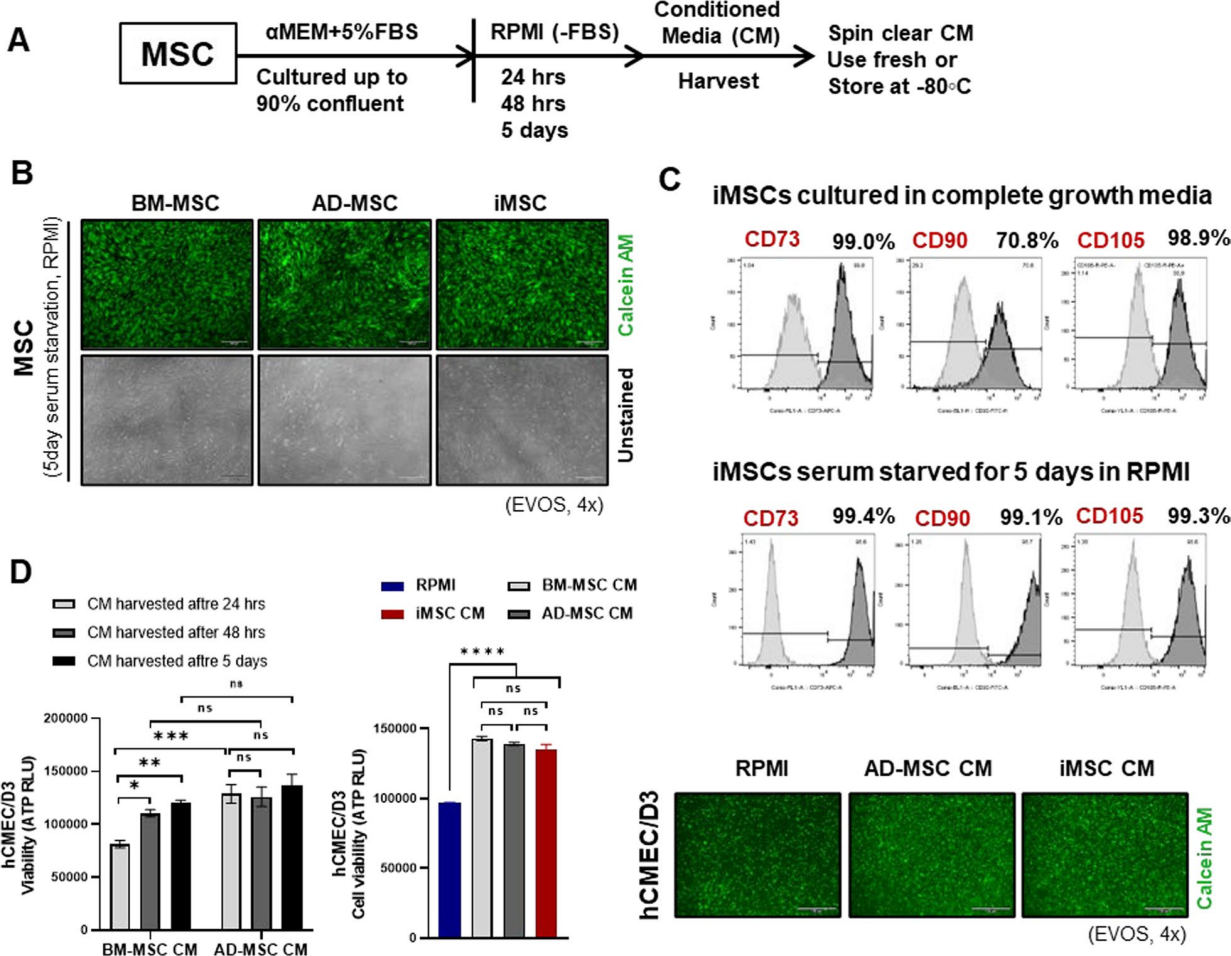
Impact of the MSC secretome on vascular endothelial cells: **A**, Scheme for MSC CM preparation. **B**, The viability and morphology of the iMSCs, BM-MSCs and AD-MSCs were assessed by staining with calcein AM 5 days after starvation in serum-free media for CM preparation. **C**, The FACS analysis of the expression of MSC markers (CD73, CD90, and CD105) was performed 5 days after starvation. **D**, hCMEC/D3 cells were cultured in MSC CM for 24 h, 48 h, or 5 days. Cell viability was assessed by measuring ATP production and calcein AM staining 24 h after CM treatment. RPMI medium was used as a control. The data are presented as the means ± SEMs (n = 5/group); *p < 0.05, **p < 0.01, ***p < 0.001, and ****p < 0.0001 according to one-way ANOVA or two-way ANOVA with Tukey’s test

**Fig. 3:**
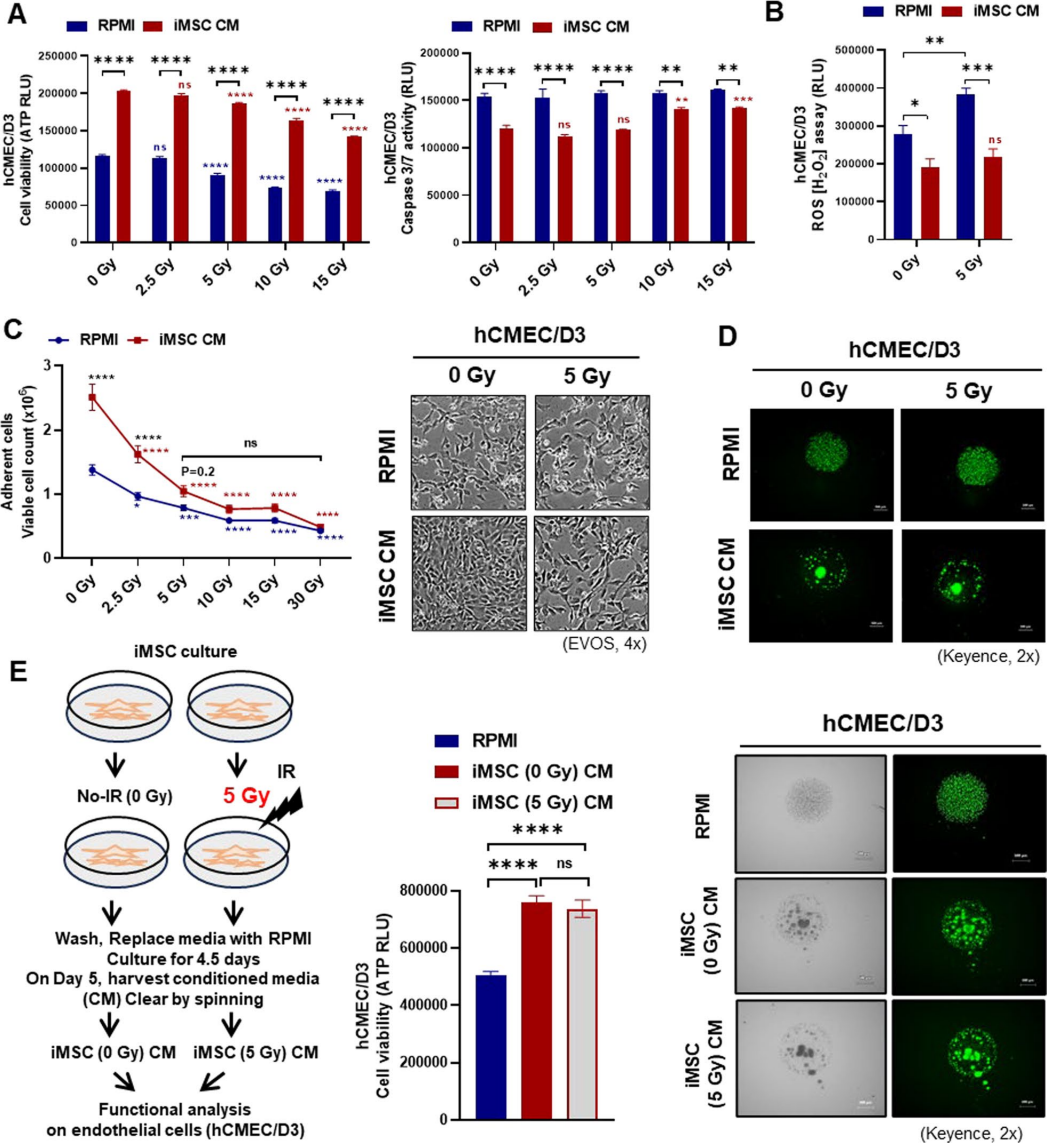
Impact of the iMSC secretome on IR-induced endothelial cell damage: **A** The viability and apoptosis of hCMECs/D3 were assessed by measuring ATP production and caspase 3/7 activity, respectively, 24 h after IR (0–15 Gy) with or without iMSC CM treatment. **B** ROS production in hCMEC/D3 cells was measured 24 h after IR at 5 Gy with or without iMSC CM treatment. **C** The adherence of hCMEC/D3 was assessed 24 h after IR (0–30 Gy) with or without iMSC CM treatment. **D** Spheroid compactness of hCMEC/D3 assessed using calcein AM staining 24 h after IR at 5 Gy with or without iMSC CM treatment. **E** CM was collected from iMSCs with or without IR at 5 Gy after conditioning for 5 days. The viability of hCMEC/D3 was assessed by measuring ATP production and spheroid compactness 24 h after iMSC CM treatment with or without IR preadministration. RPMI medium was used as a control. The data are presented as the means ± SEMs (n = 5/group); *p < 0.05, **p < 0.01, ***p < 0.001, and ****p < 0.0001 according to one-way ANOVA or two-way ANOVA with Tukey’s test

AD-MSCs, BM-MSCs, and iMSCs were starved in serum-free basal RPMI medium, and the CM was harvested sequentially after 24 h, 48 h, and 5 days to compare the potency of the MSC secretomes from different sources (Fig. 2A). The viability and retention of cell surface marker expression of iMSCs were not affected by serum starvation for 5 days (Fig. 2B, C). All subsequent experiments were performed using iMSC CM after conditioning for 5 days, as the effects of the iMSC CM and tissue-derived MSC CM on hCMEC/D3 viability were comparable (Fig. 2D). In hCMEC/D3 irradiated with various doses (0–15 Gy), cell viability was ameliorated by iMSC CM treatment, with a concomitant reduction in caspase 3/7-mediated apoptosis, irrespective of the radiation dose (Fig. 3A). While reactive oxygen species (ROS) levels were significantly elevated by IR at 5 Gy in hCMEC/D3, IR-induced ROS production was suppressed by iMSC CM treatment (Fig. 3B, Supplementary Fig. 4A). hCMEC/D3 were cultured as monolayers and irradiated with various doses (0–30 Gy) to evaluate the effect of iMSC CM on endothelial cell–substrate adhesion. IR reduced hCMEC/D3 adherence in an IR dose-dependent manner. The IR-induced reduction in cell adherence was also ameliorated by the administration of iMSC CM (Fig. 3C, Supplementary Fig. 4B). Next, we investigated the effects of IR and iMSC CM on hCMEC/D3 spheroid formation to measure cell‒cell adherence. We observed that hCMEC/D3 cells formed compact spheroids in the presence of complete endothelial growth medium (EndoGro and EGM2); however, a 5 Gy IR dose was sufficient to disrupt spheroid compactness, thus weakening cell–cell contacts. While no spheroids could form in the presence of RPMI media, hCMEC/D3 spheroids formed when cultured in iMSC CM and were more compact, irrespective of the dose of IR administered. Calcein AM/BOBO3i (Live/Dead) staining revealed fewer BOBO3i-positive dead cells in the presence of iMSC CM than in the presence of RPMI CM, indicating the protective effects of the iMSC secretome on cell death (Fig. 3D, Supplementary Fig. 4C). We also evaluated whether the effect of the secretome obtained from irradiated iMSCs was comparable to that of the secretome obtained from nonirradiated iMSCs. We observed a significant increase in cell viability and compactness in hCMEC/D3 spheroids in the presence of iMSC CM, irrespective of IR preadministration to iMSCs (Fig. 3E).

### The iMSC secretome facilitates endothelial tube morphogenesis and angiogenesis

**Fig. 4:**
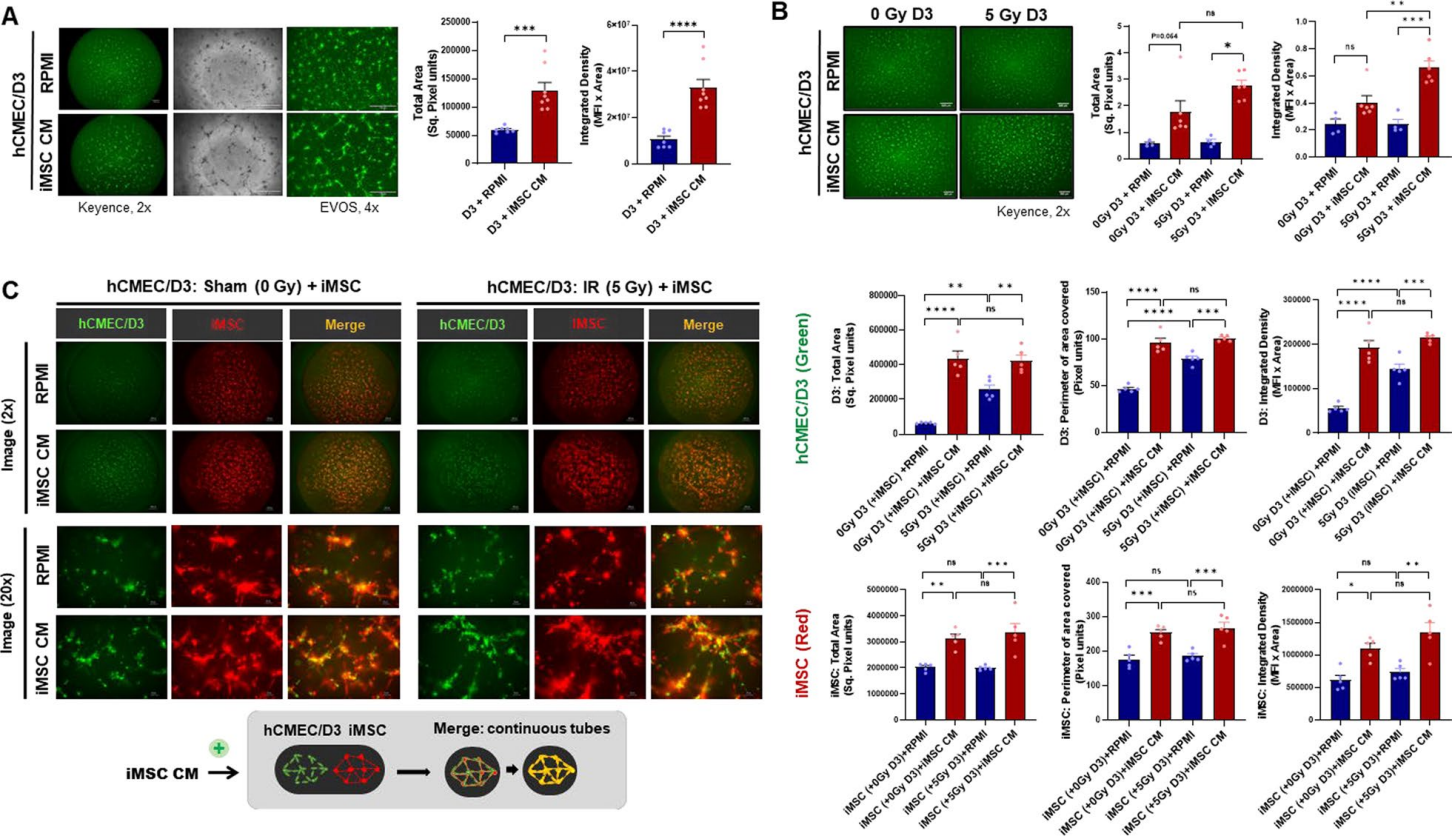
Facilitated endothelial cell tube formation by the iMSC secretome: **A** Angiogenic sprout formation of hCMEC/D3 was assessed 24 h after culture on BME-precoated 96-well plates in the presence of iMSC CM. **B** hCMEC/D3 cells (30,000 cells/well) were irradiated with 5 Gy and cultured on BME-precoated 96-well plates for 24 h in the presence or absence of iMSC CM. Angiogenic sprouting and endothelial cell tube formation were assessed by performing calcein AM staining 24 h after iMSC CM treatment. **C** hCMEC/D3 cells (20,000 cells/well) were cultured on precoated 96-well plates for 24 h and irradiated with 5 Gy. The morphology was assessed 6 h after coculture with iMSCs (10,000 cells/well) with or without iMSC CM treatment. hCMEC/D3 and iMSCs were stained with calcein AM (green) and CellTracker Red, respectively. hCMEC/D3 (20,000 cells/well) and iMSCs (10,000 cells/well) were cocultured on precoated 96-well plates for 24 h. The morphology was assessed 24 h after iMSC CM treatment. RPMI medium was used as a control. The total area, perimeter and integrated density of the green and red signals in the respective images were quantified using ImageJ. The data are presented as the means ± SEMs (n = 5/group); *p < 0.05, **p < 0.01, ***p < 0.001, and ****p < 0.0001 according to one-way ANOVA or two-way ANOVA with Tukey’s test

The impact of iMSC CM on angiogenesis was assessed through an endothelial tube formation assay in hCMEC/D3 cells cocultured with or without iMSCs. We found that iMSC CM caused pronounced hCMEC/D3 adherence and spreading, as well as the formation of angiogenic sprouts and short endothelial tubes (Fig. 4A, B, Supplementary Fig. 5A). CM collected from iMSCs irradiated with 5 Gy preserved the proangiogenic effect on hCMEC/D3. Additionally, iMSC CM stabilized the endothelial tube network, irrespective of the radiation status of the iMSCs, but the network started to disintegrate within 6 h of treatment (Supplementary Fig. 5B, C). Although we only observed short tubes formed by endothelial cells after CM treatment, iMSC CM treatment facilitated more complete tube network formation in the iMSC monoculture. Therefore, we next evaluated whether coculturing iMSCs and endothelial cells would enhance endothelial tube formation. Coculturing hCMEC/D3 with iMSCs indeed led to the formation of angiogenic tube meshwork by 6 h, which was better retained for up to 36 h in the presence of iMSC CM. hCMEC/D3 cells were stained green with calcein AM, irradiated at 0 Gy or 5 Gy, and cocultured with CellTracker Red-stained iMSCs in the presence of iMSC CM to determine the dynamics of tube formation. Intriguingly, a prominent meshwork of tubes started to be formed by iMSCs, and endothelial cells followed the path of the iMSC meshwork, which was augmented in the presence of iMSC CM. After 24–36 h of coculture, hCMEC/D3 cells and iMSCs aligned perfectly to one another at both nodes and angiogenic tubes in the presence of iMSC CM (Fig. 4C, Supplementary Fig. 5E, F). The amounts of analytes in the iMSC CM were not affected by IR administration (Supplementary Fig. 6).

**Fig. 5:**
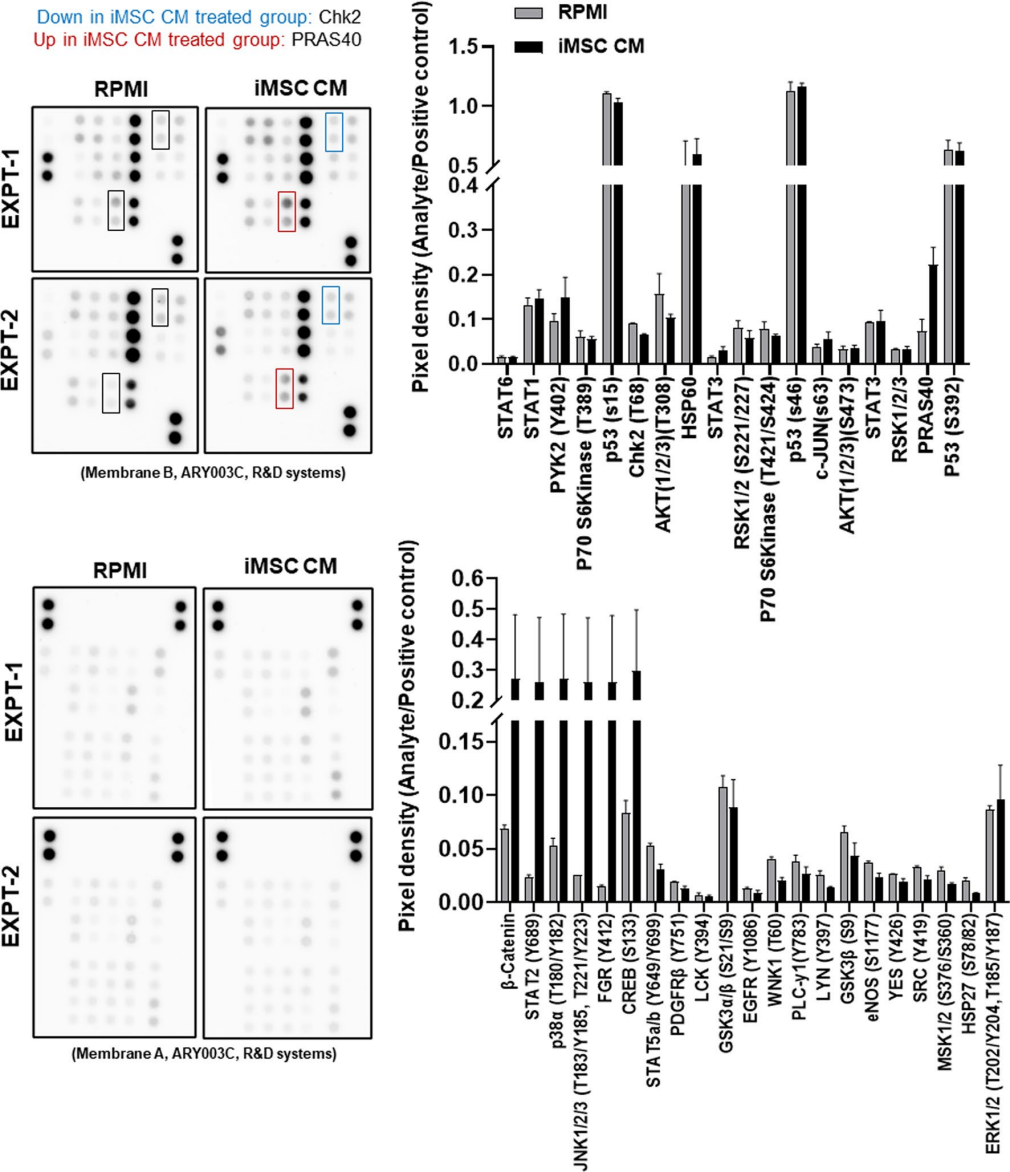
Kinome analysis of endothelial cell signalling pathways impacted by the iMSC secretome: hCMEC/D3 cells were treated with RPMI medium or iMSC CM for 24 h, followed by human kinome profiling. Dot blots of two independent experiments and their pixel density analysis are shown. RPMI medium was used as a control. The data are presented as the means ± SEMs (*n* = 2/group)

**Fig. 6:**
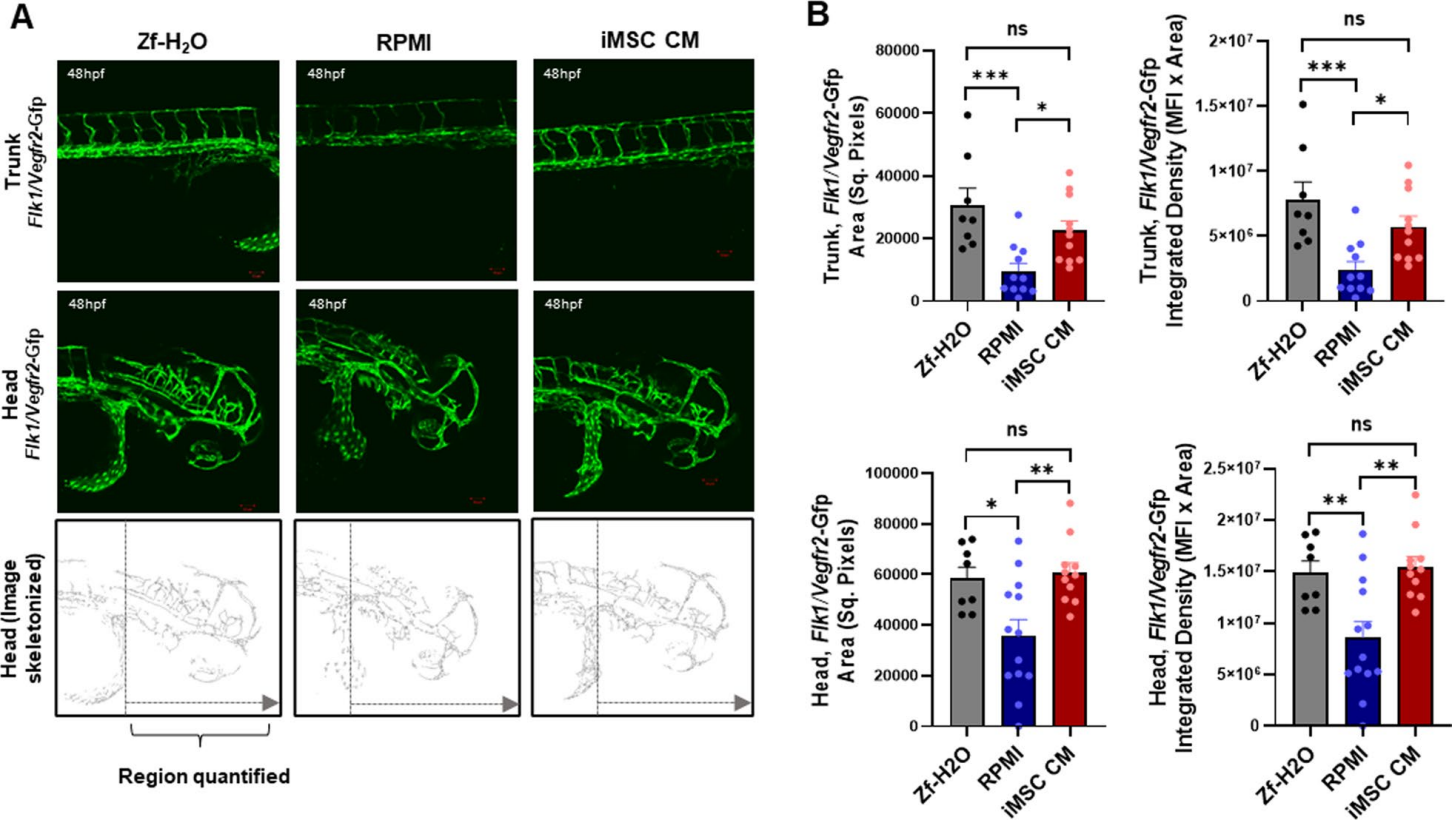
Proangiogenic effects of the iMSC secretome in vivo: **A** A *Tg*(*flk1:EGFP*) zebrafish model expressing green fluorescent protein (GFP) driven by the promoter of the *flk1/vegfr2* gene was utilized. The zebrafish embryos were harvested at 24 h postfertilization (hpf), dechorionated, and incubated with zebrafish water (Zf-H_2_O), RPMI medium or iMSC CM. After 24 h of treatment (48 hpf), the expression of GFP in the head and trunk regions of the embryos was assessed using confocal microscopy. **B** The total GFP-positive area and the integrated signal density were quantified using ImageJ. The data are presented as the means ± SEMs (*n* = 8–13 embryos/group); *p < 0.05, **p < 0.01, and ***p < 0.001 according to one-way ANOVA with Tukey’s test

hCMEC/D3 cells were treated with or without iMSC CM and subjected to a phosphokinome analysis to identify changes in signalling pathways impacted by iMSC CM. A trend towards increased phosphorylation of PYK2 and PRAS40 was observed after iMSC CM treatment. PYK2 and PRAS40 are related to the focal adhesion kinase (FAK) and PI3K3/mTOR pathways, respectively. We also found that iMSC CM suppressed the phosphorylation of the DNA damage response protein Chk2 in hCMEC/D3 cells. Additionally, the expression of regulators of the Wnt/β-catenin pathway and stress response pathways tended to increase in the presence of iMSC CM (Fig. 5). Furthermore, the impact of iMSC CM on angiogenesis in vivo was assessed using a *Tg(flk1-EGFP*) zebrafish model expressing EGFP under the control of the promoter of the vascular endothelial cell marker *flk1/vegfr2* gene. Culturing the dechorionated zebrafish embryos in iMSC CM for 24 h led to the retention of the normal morphology of the head and trunk region and significant EGFP expression which was comparable with that observed in the natural growth environment, Zf-H_2_O, but compromised in the presence of RPMI media. The figure represents data from two independent experiments, both having shown significantly higher *flk1/vegfr2*: EGFP expression in head and trunk region (p < 0.05) for iMSC CM treatment group versus RPMI, and no significant change between Zf-H_2_O and iMSC CM treatment, indicating ability of iMSC secretome to facilitate near normal angiogenesis in vivo (Fig. 6).

### The iMSC secretome contains proangiogenic and immunosuppressive factors

We explored the composition of the iMSC secretome with a targeted approach utilizing dot blot membranes spotted with various proangiogenic or antiangiogenic and immunomodulatory cytokines. The array revealed that iMSC CM contains 7 consistently abundant cytokines, chemokines and growth factors, which include monocyte chemotactic protein-1 (MCP-1, CCL2), interleukin-6 (IL6), interleukin-8 (IL-8, CXCL8), angiogenin (ANG), Gro/melanoma growth stimulating activity (Gro/MGSA), Gro-alpha, CXCL1 (RANTES), regulated upon activation, normally T-expressed, and presumably secreted, CCL5), and stromal cell-derived factor 1 (SDF-1) (Table S5). The patterns of analytes in the AD-MSC CM and BM-MSC CM were largely similar, with IL6 and MCP1 being prominent molecules. We observed a greater co-occurrence of BDNF and HGF in iMSC CM than in AD-MSC CM or BM-MSC CM (Supplementary Fig. 7A, B). We did not observe evident differences in endothelial cell tube formation between AD-MSC CM and iMSC CM (Supplementary Fig. 7C).

**Fig. 7:**
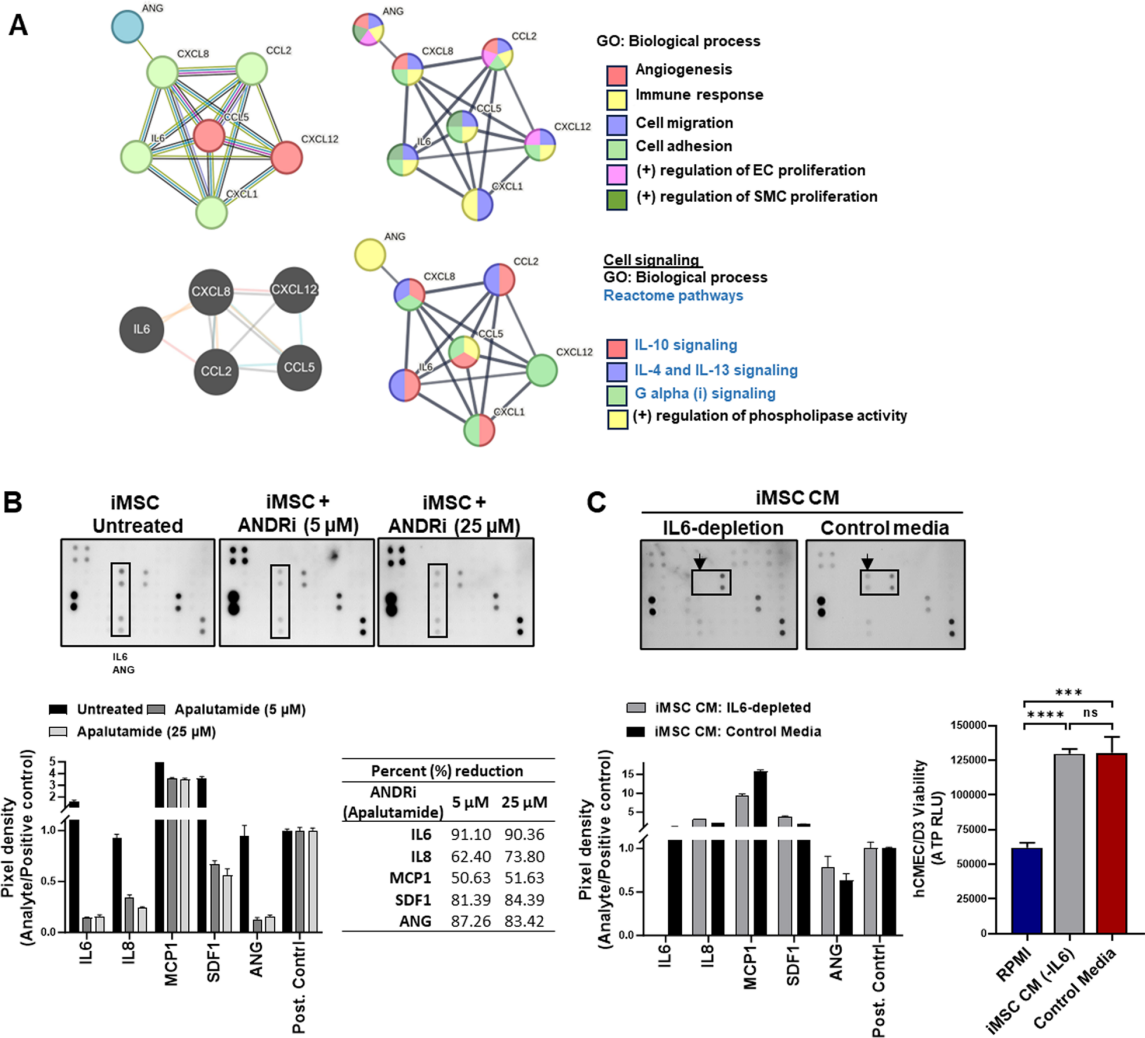
Targeted analysis of the proangiogenic factors in the iMSC secretome: **A** The interactomes of the main secreted molecules (MCP1/CCL2, IL6, IL8/CXCL8, ANG, GROα/CXCL1, RANTES/CCL5, and SDF1/CXCL12) detected in iMSC CM were obtained from the STRING database (upper left panel), and Pathway Commons (lower left panel) were identified. Functional annotation was performed using the STRING database for GO biological process terms (black) and Reactome pathways (blue). The associations of individual molecules with selective functions are indicated by colour-coded circles. **B** iMSCs were treated with the androgen receptor inhibitor (ANDRi; apalutamide) at different concentrations (0, 5, and 25 µM) for 5 days, and the levels of IL6, IL8, MCP1, SDF1, and ANG in the iMSC CM were assessed using dot blotting. Estimation of percent reductions in signal intensity of each molecule are shown. C, IL6 was specifically eliminated from iMSC CM using antibody-conjugated beads. The effects of IL6-depleted iMSC CM (-IL6) and neat iMSC CM (control) on hCMEC/D3 viability were assessed by measuring ATP production 24 h after treatment. RPMI medium was used as a control. The data are presented as the means ± SEMs (n = 4–5/group); ***p < 0.001 and ****p < 0.0001 according to one-way ANOVA with Tukey’s test

The STRING database was utilized to functionally annotate the factors identified in the iMSC secretome (Supplementary Excel sheet 1). Based on the clustering analysis, *ANG* was associated with IL8 (*CXCL8*) in the cluster of analytes containing *CXCL8*, *IL6*, and MCP-1 (*CCL2*). RANTES (*CCL5*) and SDF-1 (*CXCL12*) formed an independent cluster, with RANTES (*CCL5)* more closely associated with *CXCL8*; thus, *CXCL8* was at the forefront of various interconnected clusters. The interaction of *IL6* with *CCL2* for coexpression (red line) was verified by the Pathway Commons database, where connections are colour coded as red (coexpression), blue (binding), orange (modification) and grey (other), thus explaining the coproduction of *IL6* and *CCL2* in all conditioned media, irrespective of the cell source and time point of harvest. Based on a Gene Ontology search (GO biological process), *ANG*, *CXCL8,* and *CCL2* were annotated for angiogenesis, and all analytes were associated with immunomodulation. Reactome pathway analysis further revealed the associations of *CCL2*, *IL6*, and *CXCL8* with the IL-10, IL-4, and IL-13 signalling pathways; Gro-α (*CXCL1*) and RANTES (*CCL5*) are associated with IL-10 signalling, which accounts for the induction of an immunosuppressive phenotype (Fig. 7A).

We evaluated the transcription factor (TF) binding motifs in the promoter regions (-5000 bp to + 1000 bp) of 6 molecules detected in iMSC CM (MCP1, IL6, IL8, ANG, GRO-alpha, and RANTES) utilizing tools available in the MEME suite 5.5.4. DAL80 (yeast GATA factor), androgen receptor (ANDR), interferon regulatory factor (IRF1), zinc finger protein (ZNF) and TFAP2 transcription factor (TFAP2A/C) were identified as the top-ranked TF-binding motifs. By identifying the common TF-binding sites in the 3–4 kb promoter region using Swiss Regulon, we further confirmed the presence of several TFAP2 binding motifs, along with those for other TFs (SP/KLF and TBX). We therefore further investigated the frequency of occurrence of these common TF motifs identified through the MEME suite 5.5.4 in the promoter regions of these 6 molecules using the GTRD database. ANDR binding sites were the most abundant among the other TF motifs, with at least ≥ 18 binding sites in the promoters of all 6 molecules, followed by ≥ 8 GATA2 binding sites in each promoter. The prevalence of common regulatory elements suggested that the transcription of these 6 molecules in the iMSC secretome was coregulated by one or more of the top-ranked transcription factor-binding motifs (Table S6, Supplementary Excel sheet 2). We treated iMSCs with the androgen signalling inhibitor (ANDRi) apalutamide (RPMI medium) at concentrations of 5 μM and 25 μM to validate the comodulatory effect of the androgen cascade on the expression of analytes in the iMSC secretome. CM were harvested on Day 5 and analysed for the presence of secretome factors using dot blotting. We observed a general trend towards a decrease in the signal intensity for analytes in ANDRi-treated iMSC CM, as opposed to that in control untreated CM (Fig. 7B). These results indicate that ANDR signalling is a major pathway regulating the production of therapeutic factors in iMSCs. IL6 showed a maximal percent reduction in signal intensity, followed by ANG, IL8, and MCP1. Since IL6 is a known mediator of angiogenesis and immune modulation, we depleted IL6 from iMSC CM using antibody-conjugated beads. We detected significantly enhanced hCMEC/D3 viability in the presence of IL6-depleted iMSC CM, which was comparable to that in the presence of complete iMSC CM (Fig. 7B). Thus, other factors or multiple factors in iMSC CM may mediate the beneficial effects on vascular endothelial cells.

We performed RNA sequencing of iMSCs, AD-MSCs, and BM-MSCs to compare the transcriptomes of MSCs from different sources (Supplementary Excel sheet 3). We observed higher expression of genes related to rejuvenation, including *VANGL2*, *SOX11*, *IGFBP3/5,* and *PARP1*, in iMSCs than in AD-MSCs and BM-MSCs (Supplementary Fig. 8A). Although developmental transcription factors of the HOX/TBX family are also involved in the positive regulation of angiogenesis, the expression of these genes was higher in AD-MSCs and BM-MSCs than in iMSCs (Supplementary Fig. 8B). Among the biological processes upregulated in the tissue-derived MSCs compared to the iMSCs, the top 30 GO terms included pathways related to angiogenesis and tube formation. The top 30 downregulated biological processes included ribosome biogenesis, rRNA processing, translation, mitochondrial respiration, ATP biosynthesis, and pathways related to DNA damage response and repair (Supplementary Fig. 8C). According to the results of the Reactome pathway analysis, genes related to neuronal development, such as SLIT/ROBO and axon guidance, were also found to be upregulated in iMSCs (Supplementary Fig. 8D). When the expression of transcription factors was assessed, we observed that *TFAP2A*, *TFAP2C*, and *GATA2* were upregulated in iMSCs compared to AD-MSCs and BM-MSCs (Supplementary Fig. 8E). Among the genes detected in the iMSC secretome, *IL6*, *ANG*, *VEGF-A*, and *VEGF-C* were more highly expressed in AD-MSCs and BM-MSCs than in iMSCs. On the other hand, the expression of immunosuppressive cytokine/chemokine-encoding genes, including MCP1 (*CCL2*), IL8 (*CXCL8*), *TGFB2*, and Gro-α (*CXCL1*), was higher in iMSCs than in control cells (Supplementary Fig. 8F).

### The iMSC secretome has immunosuppressive effects on monocytes

NF-κB reporter activity in THP1 cells was significantly increased after IR at 10 Gy and 15 Gy and was suppressed by iMSC CM treatment (Supplementary Fig. 9A). While iMSC CM increased the viability of THP1 cells, as evaluated by ATP production, regardless of IR, the TNF-α concentration in the culture medium normalized to the cell viability was reduced by iMSC CM treatment (Supplementary Fig. 9B). Consistently, iMSC CM treatment also prevented ROS production in THP1 cells treated with or without IR (Supplementary Fig. 9C). hCMEC/D3 and/or iMSCs were irradiated at 5 Gy and cocultured with THP1-NFκB-Luc2 cells to assess the cross-talk between immune cells and IR-damaged cells. NF-kB reporter activity in THP1 cells was elevated in the presence of irradiated hCMEC/D3 cells and/or iMSCs, indicating immune cell activation by IR-damaged cells. We found that iMSC CM significantly reduced NF-kB reporter activation in THP1 cells activated by IR-damaged cells (Supplementary Fig. 9D). In addition, the clustering of THP1 cells was investigated to evaluate the activation status after stimulation. While LPS stimulation led to THP1 cell clustering, iMSC CM significantly reduced LPS-induced clustering compared to that in the RPMI control group (Supplementary Fig. 9E-G). We also investigated how the irradiated human microglial HMC3 cell line influenced THP1 cell activation in the presence or absence of iMSC CM. NF-kB activation in THP1-NFκB-Luc2 cells was significantly elevated when they were cocultured with HMC3 cells irradiated at 5 Gy in RPMI media, as opposed to when they were cocultured with nonirradiated intact HMC3 cells. Consistent with the results from hCMECs/D3 and/or iMSCs, we found that THP1-NF-κB cell activation induced by irradiated HMC3 cells was suppressed by iMSC CM treatment (Supplementary Fig. 9H).

## Discussion

Among current tumour treatment modalities, radiation therapy has been known to most effectively augment the surgical response. Due to increased awareness of the tissue toxicity caused by conventional radiation therapy, particularly cerebral arteriovenous malformations (AVMs) and a decline in the stem cell pool, further improvements in methodologies incorporating heavy ions, such as proton-beam therapy and carbon-ion therapy, are encouraged [79–82]. Although novel radiation therapies are promising approaches for eliminating tumour masses, sufficient information on their long-term benefits has not yet been obtained. The availability of these materials is also limited due to their high-cost infrastructure and expense of treatment [83, 84]. With the improved 5-year median survival of > 60% in patients with malignant brain tumours and > 90% in patients with nonmalignant brain tumours, identifying regenerative commodities to restore homeostasis and brain health in long-term cancer survivors has become imperative [85, 86]. MSCs have emerged as promising regenerative therapeutics. MSCs possess multiple properties, including weak immunogenicity, tumour trophism, the ability to cross the BBB, the ability to trace glioma microsatellites, regenerative capacity, immunomodulatory potential, proangiogenic properties and the possibility of being rapidly cleared naturally or by synthetic biological means. Together, these attributes have spurred investigations of MSCs for an array of applications in neurology and neurosurgical oncology [54–56, 87–94]. While some controversies and challenges are associated with cell-based therapies, MSCs can be orchestrated to produce a selective composite of rejuvenating factors by applying various stimuli, including radiation, which makes them ideal candidates for cell-free therapies to treat various degenerative diseases [62, 63, 95–99]. Studies have shown a protective role of stem cell therapies in radiation-induced brain injury [38, 100]; however, our knowledge about the therapeutic potential of the iMSC secretome in treating RIBI is limited.

In our in vitro experiments on radiation-induced cell injury, the iMSC secretome increased cell survival, adhesion, spreading, migration, and morphogenesis in the human brain vascular endothelial cell line hCMEC/D3. We confirmed the same therapeutic effects using iMSCs differentiated from another independent iPSC line (Supplementary Fig. 10). We performed angiogenic tube formation assays in vitro to evaluate the proangiogenic effects of the iMSC secretome. Our results showed that coculture of hCMEC/D3 cells and iMSCs facilitated continuous meshwork formation in endothelial cell tubes in the presence of iMSC CM. Additionally, the iMSC secretome augmented endothelial cell function likely by upregulating pathways mediated by PYK2, PIK3/mTOR and Wnt. Notably, MSCs have some overlapping properties with pericytes, which play a critical role in endothelial cell network formation both in vitro and in vivo [114, 115]. Since pericytes are known to mediate cerebrovascular integrity and neuroregeneration [116, 117], a plausible hypothesis is that the iMSC secretome could complement and augment the effects of iMSC-based therapy on vasculature regeneration and repair. The iMSC secretome also suppressed ROS production and NF-κB-mediated proinflammatory phenotypes in the human monocyte THP1 cell line. NF-κB predominantly mediates inflammatory signalling in mononuclear cells and monocytes through the release of proinflammatory cytokines in response to tissue stressors [101–103]. During CNS injury, NF-κB-dependent release of TNF-α from monocytes or microglia exacerbates vascular damage by increasing ROS levels and disrupting the BBB [104–107]. Thus, the suppression of the NF-κB pathway in monocytes by iMSC secretome treatment could lead to beneficial effects on RIBI. Several studies have shown that factors in the MSC secretome, such as MCP1, IL6, and IL8, induce immunosuppressive phenotypes by promoting the M2 polarization of monocytes and by recruiting myeloid-derived suppressor cells and regulatory T cells [64–66, 108]. A recent study by Reina et al. showed that MSC-conditioned media can upregulate genes involved in antioxidant defences in zebrafish, thereby alleviating ROS-mediated tissue damage [109]. Consistent with these findings, we observed a reduction in the levels of ROS produced by endothelial cells and THP1 monocytes in response to irradiation. Increased ROS levels and vascular damage are observed during tumour progression, as well as during radiation therapy [110, 111]. As stem cell-based approaches have been evaluated for regenerative therapy for various chronic disorders, our results reveal the promising ability of the iMSC secretome for tissue repair by ameliorating IR-induced ROS production and vascular damage. Cerebrovascular damage is involved in the pathogenesis of neurodegenerative disorders, including Alzheimer’s disease [14, 15] and cancer-related cognitive impairment [112, 113]. We also detected BDNF in iMSC CM, which could have direct implications for neuroregeneration. Therefore, a plausible hypothesis is that regenerative therapies using the iMSC secretome may also alleviate the pathophysiology of not only RIBI but also age-related neurodegenerative diseases.

A cytokine-based antibody array revealed proangiogenic and immunosuppressive factors in the iMSC secretome that could contribute to these observations. Although our study was limited to targeted panels for angiogenesis and immunomodulation, we identified several cytokines, chemokines, and growth factors that were consistently detected in the secretomes of iMSCs, BM-MSCs, and AD-MSCs, including MCP1, IL6, and IL8. We confirmed the composition and functional relevance of the iMSC secretome by utilizing at least two independent iMSC lines and confirmed that MCP1, IL6 and IL8 were the top three secreted factors. We further showed the correlations of these three molecules with IL10 signalling, a known anti-inflammatory cascade, based on protein interactome and gene correlation analyses. Although IL6 is a key cytokine, depleting IL6 from the iMSC secretome did not impact the prosurvival effect of iMSC CM on endothelial cells. This observation implies that other factors in IMSC CM, such as MCP1, IL8, and ANG, may play a predominant role in mediating the therapeutic effects. Additionally, the functional attribute of the iMSC secretome is likely not a property of one factor alone but rather a composite outcome of multiple factors with overlapping functions. In addition, we investigated the promoter regions of the molecules and identified common binding sites for transcription factors such as ANDR, GATA2, and TFAP2A/C, with highest binding motif frequency observed for the ANDR. Thus, these transcription factors may modulate the extracellular release of soluble proteins that share proangiogenic and immunosuppressive properties. ANDR is known to modulate cerebrovascular unit formation and angiogenesis [118, 119]. GATA2 has been shown to be the primary regulator of the immunosuppressive phenotype observed in young MSCs [120]. Consistently, we showed that the IL6, IL8, ANG, and MCP1 levels in iMSC CM were substantially reduced upon administration of an androgen receptor signalling inhibitor. Thus, ANDR signalling predominantly mediates the production of these factors in iMSCs. Although further studies are necessary, activating ANDR may improve the therapeutic efficacy of iMSCs.

Irrespective of IR administration to iMSCs, we observed a proangiogenic and immunosuppressive signature in the iMSC secretome, which is consistent with previous reports showing that MSCs can better resist radiation stress and retain their functional properties [121, 122]. Although enhanced DNA damage repair has been documented for tissue-derived MSCs [123, 124], our RNA sequencing results revealed the upregulation of biological processes related to DNA damage repair, rRNA processing and mitochondrial function in iMSCs compared to tissue-derived MSCs. Therefore, iMSCs may be more resilient to radiation stress and be more rejuvenating than tissue-derived MSCs. However, the use of a high IR dose of 30–60 Gy to treat tumours may still partly impair the functions of tissue-resident MSCs or engrafted MSCs and may influence their secretome [125]. These observations indicate the applicability of the iMSC and iMSC secretomes as potential therapeutic products for treating RIBI. Further studies are needed to confirm these effects in vivo, and further optimization of the concentration of conditioned media may facilitate the development of a potent therapy-grade secretome for biotherapeutic applications against radiation-induced brain injury.

In summary, our study provides a new paradigm for the development of iMSC secretome-based therapeutics for brain damage caused by radiation therapy, accidental radiation spillage, radiation-based warfare [126], and space radiation-induced neurocognitive impairment in astronauts [127–131]. We are constantly exposed to galactic radiation reaching our atmosphere, and together with elevated levels of atmospheric pollutants, neurodegeneration can be exacerbated [132]. Molecular neuroprotective drugs can have off-target effects that can add complexity to the treatment regime of chemoradiation therapy [133, 134]. Cell biotherapeutics are therefore gaining attention, and iMSC-based secretome therapies can have advantages over cell therapies [135]. However, one of the present limitations with iPSC technology is the cost involved in manufacturing and quality control assessments for a GMP-grade biotherapeutic product for mass application. Therefore, the identification of strategies to optimize product production and augment the tissue regeneration capabilities of iMSCs both in vitro and in vivo, such as by synergizing them with natural supplements, antioxidants, and neuroprotective compounds, is imperative [136–138]. Optimizations of manufacturing procedures are required to establish a more potent therapy-grade secretome for biotherapeutic applications for RIBI in a radiation treatment dose-dependent manner. Secretome from engineered MSCs with elevated expression of transcription factors such as ANDR or GATA2 may also potentiate their therapeutic effects on RIBI. MSCs are being evaluated for their potential to eradicate glioma stem cells and eliminate tumour masses by synergizing with RT; however, limitations and risks exist due to their prolonged persistence at the site of the resected tumour or in impacted tissue [54]. Importantly, MSCs can exhibit both proinflammatory and immunosuppressive abilities, depending on the microenvironment in which they are present or implanted [55, 56]. Since a rejuvenating MSC secretome can also be protumourigenic and can trigger quiescent cancer stem cells to proliferate and form secondary foci, the application of iMSC-based therapies to combat RIBI in posttreatment cancer care or long-term cancer survivorship should be considered with caution. Reports suggest adopting approaches to ensure the clearance of spent MSCs after tumour treatments and, for radioprotective and regenerative purposes, to defer their administration until complete remission of residual disease is achieved to eliminate the possibility of tumour recurrence [90, 91, 139–142].

## Conclusions

Our results indicate that iMSCs produce proangiogenic and immunosuppressive factors with a signature of analytes comprising MCP1, IL6, IL8, and ANG, along with other factors, which collectively act to alleviate radiation-induced vascular damage and immune activation. Thus, iMSC secretome treatment may ameliorate radiation-induced bystander effects during RIBI and induce radioprotection and tissue regeneration in long-term cancer survivors (Supplementary Fig. 11).

### Supplementary Information

Supplementary file 1.

## Data Availability

The RNA sequencing dataset supporting the conclusions of this article is included within the article (Excel sheets, Supplementary materials). This data has been submitted to GEO repository for public availability with, GEO accession number, GSE271374.

## Abbreviations

RT: Radiation therapy
IR: Irradiation
LDIR: Low-dose irradiation
RIBI: Radiation-induced brain injury
ROS: Reactive oxygen species
CNS: Central nervous system
AD: Alzheimer’s disease
VCID: Vascular cognitive impairment and dementia
aMSC: Mesenchymal stem cell
AD-MSC (ADSC): Adipose-derived mesenchymal stem cell
BM-MSC: Bone marrow-derived mesenchymal stem cell
TD-MSC: Tissue-derived mesenchymal stem cell
iPSC: Induced pluripotent stem cell
iPSC-MSC (iMSC): Induced pluripotent stem cell-derived MSC
EC: Endothelial cell
CRCI: Cancer-related cognitive impairment
GBM: Glioblastoma
LGG: Low-grade glioma
TF: Transcription factor
CM: Conditioned media
MCP1: Monocyte chemotactic protein 1
IL6: IL8, IL10, Interleukin 6, Interleukin 8, Interleukin 10
IL2: IL4, IL13, Interleukin 2, Interleukin 4, Interleukin 13
RANTES: Regulated upon activation, normally T-expressed, and presumably secreted
SDF1: Stromal cell-derived factor 1
TGF-β: Transforming growth factor-beta
NF-κB: Nuclear factor kappa-light-chain-enhancer of activated B cells
TNF-α: Tumour necrosis factor-alpha
CCL: Chemokine ligand
CXCL: Chemokine (C-X-C motif) ligand
ANG: Angiogenin
VEGF: Vascular endothelial growth factor
CLDN5: Claudin-5
OCLN: Occludin
TJP1: Tight junction protein-1
CDH5: Cadherin-5
VCAM1: Vascular cell adhesion protein 1
ICAM1: Intercellular adhesion molecule 1
ICAM2: Intercellular adhesion molecule 2
PECAM1: Platelet and endothelial cell adhesion molecule 1
ANDR: Androgen receptor
GATA2: GATA-binding factor 2
TFAP2: Transcription factor AP-2
ZNF: Zinc-finger motif
HOX: Homeobox domain
TBX: T-box *domain*
FOXP3: Forkhead box P3
CTLA4: Cytotoxic T-lymphocyte associated protein 4
CCR8: C–C motif chemokine receptor 8
TNFRSF9: TNF receptor superfamily member 9
BDNF: Brain-derived neurotrophic factor
SLIT/ROBO: Slit guidance ligand/Roundabout guidance receptor
VANGL2: VANGL planar cell polarity protein 2
IGFBP: Insulin-like growth factor binding protein
SOX: SRY-Box transcription factor
Chk2: Checkpoint kinase 2
PYK2: Protein tyrosine kinase 2
PI3K: Phosphatidylinositol-4,5-bisphosphate 3-kinase
Akt: AKT serine/threonine kinase 1
mTOR: Mammalian target of rapamycin
Wnt: Wingless-related integration site
D3: HCMEC/D3 (human brain endothelial cell line)
Gy: Gray (unit of radiation dose, 1 Gy = absorbed dose of 1Joule/kg)
ExPASy: Expert Protein Analysis System
STRING: Search tool for the retrieval of interacting genes/proteins
GTRD: Gene transcription regulation database
GEPIA2: Gene expression profiling interactive analysis-2 (web server for large-scale expression profiling and interactive analysis)
TIMER2: Tumour immune estimation resource-2 (web server for comprehensive analyses of tumour-infiltrating immune cells)
TCGA: The cancer genome atlas
GTEx: Genotype-tissue expression database
