# Supplementary material for: Secretome from iPSC-derived MSCs exerts proangiogenic and immunosuppressive effects to alleviate radiation-induced vascular endothelial cell damage
Source: Stem Cell Res Ther. 2024 Jul 29;15:230. doi: 10.1186/s13287-024-03847-5 (PMC11287895; doi:10.1186/s13287-024-03847-5)
Supplement: Supplementary file 1 [file 13287_2024_3847_MOESM1_ESM.pdf]

Table S1. Publications on Radiation therapy and its associations
(Web of Science, 1975-2023)

Association	General (Total)		Cancer		Brain cancer	
Terminology in RT	No. of publications	Year of first mention	No. of publications	Year of first mention	No. of publications	Year of first mention
Radiation Therapy	195956	1975	123775	1975	8507	1981
X-Ray radiation therapy	6734	1976	3495	1990	210	1991
Proton beam therapy	28207	1975	7797	1991	595	1991
Boron Neutron capture therapy	3717	1975	1227	1982	291	1991
Brachytherapy	32660	1975	19273	1978	202	1991
Gamma-ray radiation therapy	1025	1980	427	1980	22	1994
Intraoperative radiation therapy (IORT)	956	1985	726	1990	15	1994
Hypofractionated Radiation therapy	3904	1989	2924	1990	150	1995
Stereotactic body radiation therapy (SBRT)	10345	1994	7390	1997	298	2000
Stereotactic radiosurgery (SRS)	3682	1994	1088	1997	911	1998
3D-conformal radiation therapy	2547	1991	2050	1991	80	1995
Intensity-modulated radiation therapy (IMRT)	8959	1997	6526	1997	264	2000
Image-guided radiation therapy (IGRT)	1122	2002	770	2002	20	2002
Volumetric modulated radiation therapy (VMAT)	2194	2008	1517	2008	105	2009
Superficial x-ray radiation therapy (SXRT)	3	2012	3	2012		
Carbon ion therapy	3384	1991	1531	1991	63	1991

Table S2. Publications on Radiation therapy association with vascular damage
(Web of Science, 1975-2023)

Association	Radiation induced vascular damage		
Terminology in RT	No. of publications	Year of first mention	~ Impact on vascular integrity (d / m)
Radiation Therapy	571	1991	d
X-Ray radiation therapy	48	1995	d
Proton beam therapy	23	1993	d / m
Boron Neutron capture therapy	8	1992	d
Brachytherapy	38	1991	d
Gamma-ray radiation therapy	4	1996	d
Intraoperative radiation therapy (IORT)	5	1991	d
Hypofractionated Radiation therapy	14	2007	d / m
Stereotactic body radiation therapy (SBRT)	17	2007	d / m
Stereotactic radiosurgery (SRS)	12	2007	d / m
Intensity-modulated radiation therapy (IMRT)	3	2002	d / m
Carbon ion therapy	1	2012	m

d, disrupts vascular integrity
m, maintains vascular integrity

Table S3. Composition of media utilized in iMSC generation

Media type, and composition				
**Induction media**				
**Reagent**	**Catalogue number**	**Stock conc.**	**Final conc.**	**For 50ml**
IMDM	Gibco; 12440053	-	1X	39.5ml
KnockOut SR	Gibco; 10828028	-	20%	10ml
Non-essential AA	Gibco; 11140050	10mM	0.1mM	0.5ml
2-Mercaptoethanol	Gibco; 21985023	55mM	0.1mM	91µl
**Differentiation media**				
**Reagent**	**Catalogue number**	**Stock conc.**	**Final conc.**	**For 50ml**
IMDM	Gibco; 12440053	-	1X	39.5ml
KnockOut SR	Gibco; 10828028	-	20%	10ml
Non-essential AA	Gibco; 11140050	10mM	0.1mM	0.5ml
2-Mercaptoethanol	Gibco; 21985023	55mM	0.1mM	91µl
TGFβ1	R&D; 240-GMP-010	100µg/ml	10ng/ml	5µl
**MSC culture media**				
**Reagent**	**Catalogue number**	**Stock conc.**	**Final conc.**	**For 500ml**
α-MEM	Gibco; 12561	-	1X	470ml
Glutamax	Gibco; 35050-061	100X	1X	5ml
FBS		-	5%	25ml

Table S4. Composition of media utilized in culture of different cell types

Cell line	Cell type	Media	FBS	Supplements
hCMEC/D3	Human brain endothelial cell line	EGM-2	+FBS (~5%)	SingleQuots™ Supplements (CC-4133, Lonza)
HMC3	Human Microglial cell line	DMEM-F12	10% FBS	Sodium pyruvate, Non-essential amino acids Glutamax, 1%Penicillin/Streptomycin
THP-1	Human monocytic cell line	RPMI	10% FBS	β-mercaptoethanol, Penecillin/Streptomycin
THP-1 NF-κB-Luc2	Human monocytic NF-κB promoter with luciferase reporter cell line	RPMI	10% FBS	β-mercaptoethanol, Puromycin, Penecillin/Streptomycin
AD-MSC	Adipose derived- MSC (ADSC)	α-MEM	5% FBS	Glutamax
BM-MSC	Bone marrow derived-MSC	α-MEM	5% FBS	Glutamax
iMSC-line 1: (iPSC-MC0039)	iPSC derived-MSC	α-MEM	5% FBS	Glutamax
iMSC-line 2 (iPSC-MC0063)	iPSC derived-MSC	α-MEM	5% FBS	Glutamax
Fibroblast (FB)	Fibroblast	DMEM high Glucose	10% FBS	Non-Essential Amino Acid , 1% Pen/Strep, 1x Amphotericin B (Gemini Bio 400-104)
All media and basic cell culture reagents were obtained from Gibco, Life Technologies, and FBS from Sigma (F0926), unless otherwise specified.				

Table S5. Analytes in iMSC secretome

**iPSC-derived MSC lines**	
**iMSC-line 1: iMSC**	
Conditioned media	iMSC CM
**Secretome iMSC ± IR:**<br>iMSC (0Gy) CM<br>iMSC (5Gy) CM	**MCP1, IL6, IL8,** ANG > GROα, RANTES, SDF1, GRO, IL10, TGFβ1
**iMSC-line 2: iMSC (L2)**	
Conditioned media	iMSC (L2) CM
**Secretome iMSC (L2) ± IR:**<br>iMSC (L2, 0Gy) CM<br>iMSC (L2, 5Gy) CM	**MCP1, IL6, IL8**, MCP3 > GROα, SDF1, ANG, GRO, IL10, RANTES, TGFβ1
**Tissue derived MSCs: TD-MSC**	
Bone marrow derived MSC: BM-MSC<br>Adipose derived MSC: AD-MSC (or ADSC)	
Conditioned media	BM-MSC CM, AD-MSC CM
**Secretome**<br>BM-MSC CM<br>AD-MSC CM	Analytes consistently detected: **MCP1, IL6 > VEGF, ANG** > SDF1<br>Analytes variably detected: IL8 > IL7, RANTES

Table S6. Regulatory elements in gene promoter of analytes identified in iMSC secretome

**A. Top-ranked TF-binding motifs in promoter region (-5000 to +1000) of prime 6 secretome factors: MCP1, IL6, IL8, ANG, GRO-alpha, RANTES**						
**TF-motif db.**	**JASPAR (non-redundant DNA)_JASPAR CORE 2022**			**HUMAN (HOCOMOCOv11)**		
Analysis Method	TF-binding Motif identified	P-value	E-value	TF-binding Motif identified	P-value	E-value
SEA	DAL80 (Yeast GATA factor)	1.08E-03	2.12E+00	ANDR	1.08E-03	4.34E-01
AME	ZNF460	5.25E-09	1.34E-03	ZN770	3.51E-10	5.26E-05
XSTREME	DOF3.6, ZNF384	1.08E-03	1.08E-03	IRF1	1.08E-03	1.08E-03
GLAM2	ZNF460<br>Other similar motif-binders: ZNF135, ZNF257, TFAP2A/B/C	1.62E-05	3.16E-05	ZN770	5.53E-9	2.22E-06
**B. Common TF-binding sites in 3-4kb promoter region of secretome factors: (by Swiss Regulon)**				**C. Frequency of TF-binding sites in promoter region of prime 6 secretome factors: (by GTRD data base):**		
SP/ KLF	SP/KLF family of transcription factors			GATA factor	GATA1<br>**GATA2**<br>GATA3<br>GATA4<br>GATA6	≥1 (1-8)<br>≥8 (8-30)<br>≥2 (2-16)<br>≥1 (1-8)<br>≥1 (1-9)
TFAP2A/C	TFAP2 family of transcription factors			ANDR	AR	≥18 (18-54)
TBL1XR1	TBX family of transcription factors			TFAP2	TFAP2A/C	≥1 (1-11)
				IRF	IRF1	≥1 (1-8)
Other TF-binding motifs present:	RUNX, Myc/Myb, STAT, NFκB, REL, HOX, FOX, AP1, IRF1, NFAT, TCF, ETS			ZNF	ZNF460<br>ZN770	NR<br>≥0 (0-3)
**Summary: Common regulatory elements identified:**	**SP/KLF, TFAP2, ZNF, TBX, GATA2, ANDR, along with other TF-binding domains present.**					

A, Top-ranked transcription factor (TF) binding motifs in promoter region (-5000 to +1000) common to the prime 6 molecules in iMSC CM (MCP1, IL6, IL8, ANG, GRO-alpha, RANTES), identified using tools available at MEME suite 5.5.4, based on the analysis methods (SEA, AME, XTREME, and GLAM2). JASPAR CORE 2022, and Human, HOCOMOCO v11 were used as Motif databases. B, Enlists common TF-binding sites in 3-4 kb promoter region of the prime 6 molecules identified by Swiss Regulon. C, Enlists the frequency of TF-binding sites for common transcription factor motifs identified in promoter region of the prime 6 molecules using GTRD database.

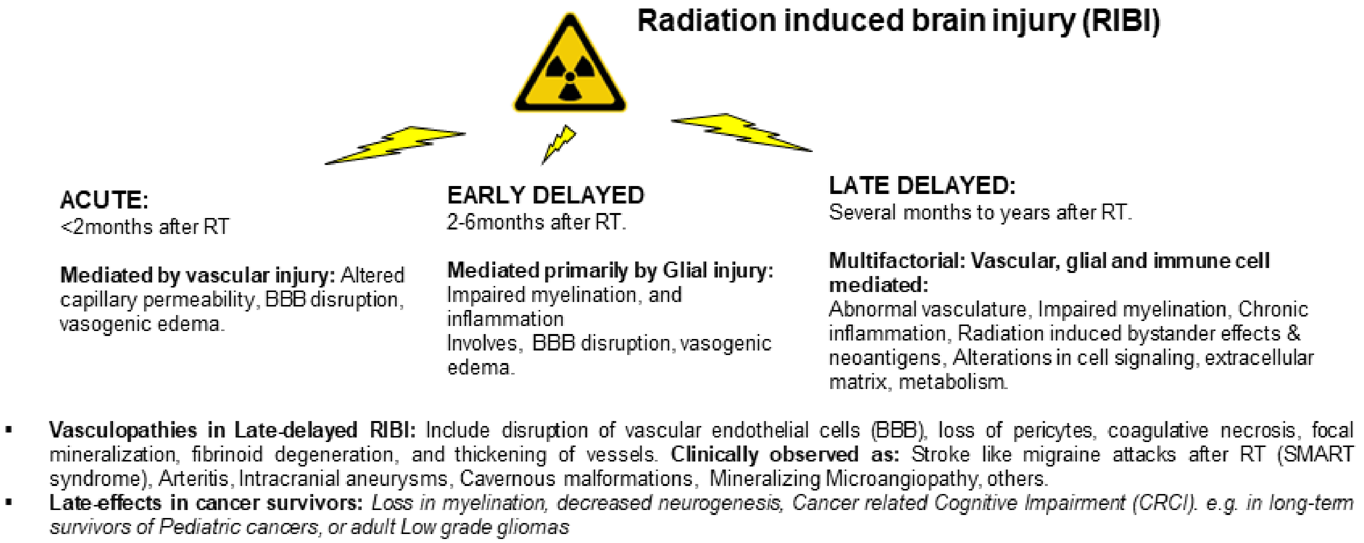

Supplementary Figure 1. Historical perspective on radiation therapy in cancer care and radiation induced vascular injury. Three phases of radiation induced brain injury and vasculopathies observed in RIBI are shown.

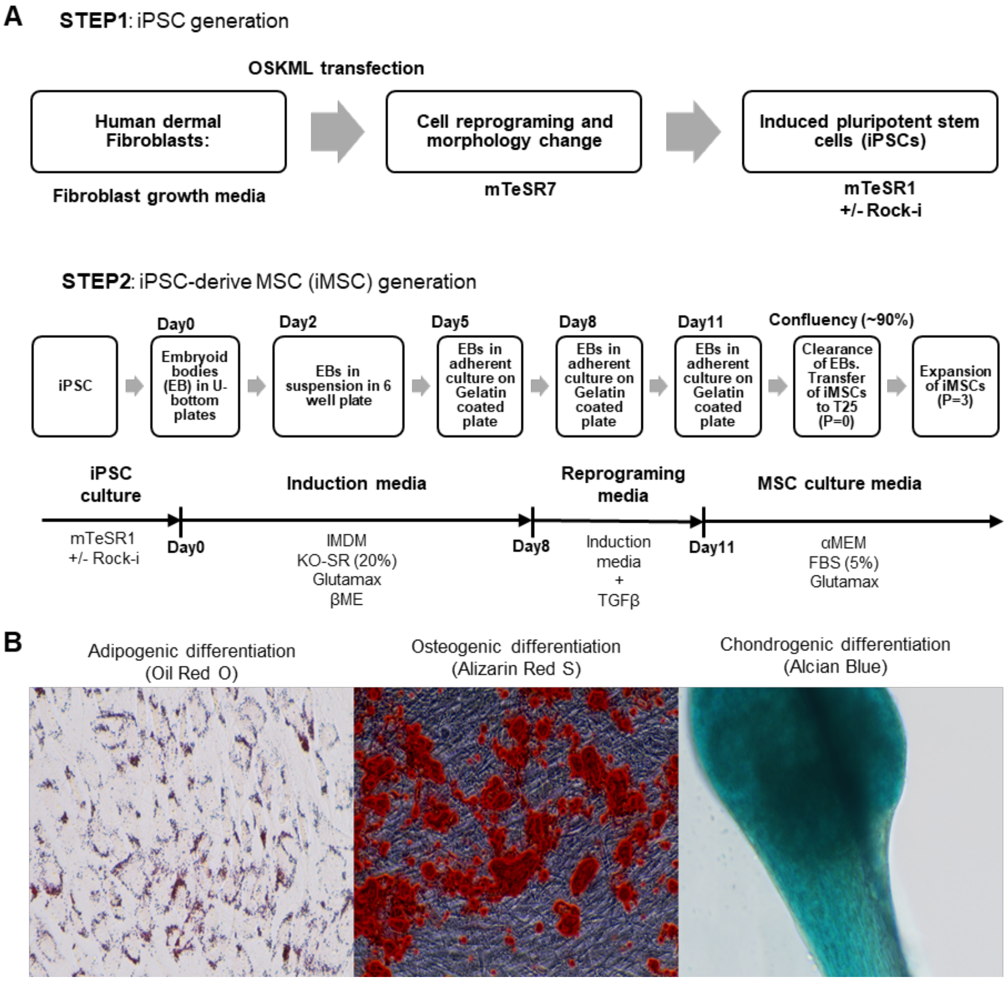

Supplementary Figure 2. A, Schematic representation of differentiation procedures of iPSCs into MSCs are shown. B, Trilineage differentiation of iMSCs to adipocytes, osteoblasts, and chondrocytes were assessed by Oil Red O staining, Alizarin Red S staining, and Alcian Blue staining, respectively.

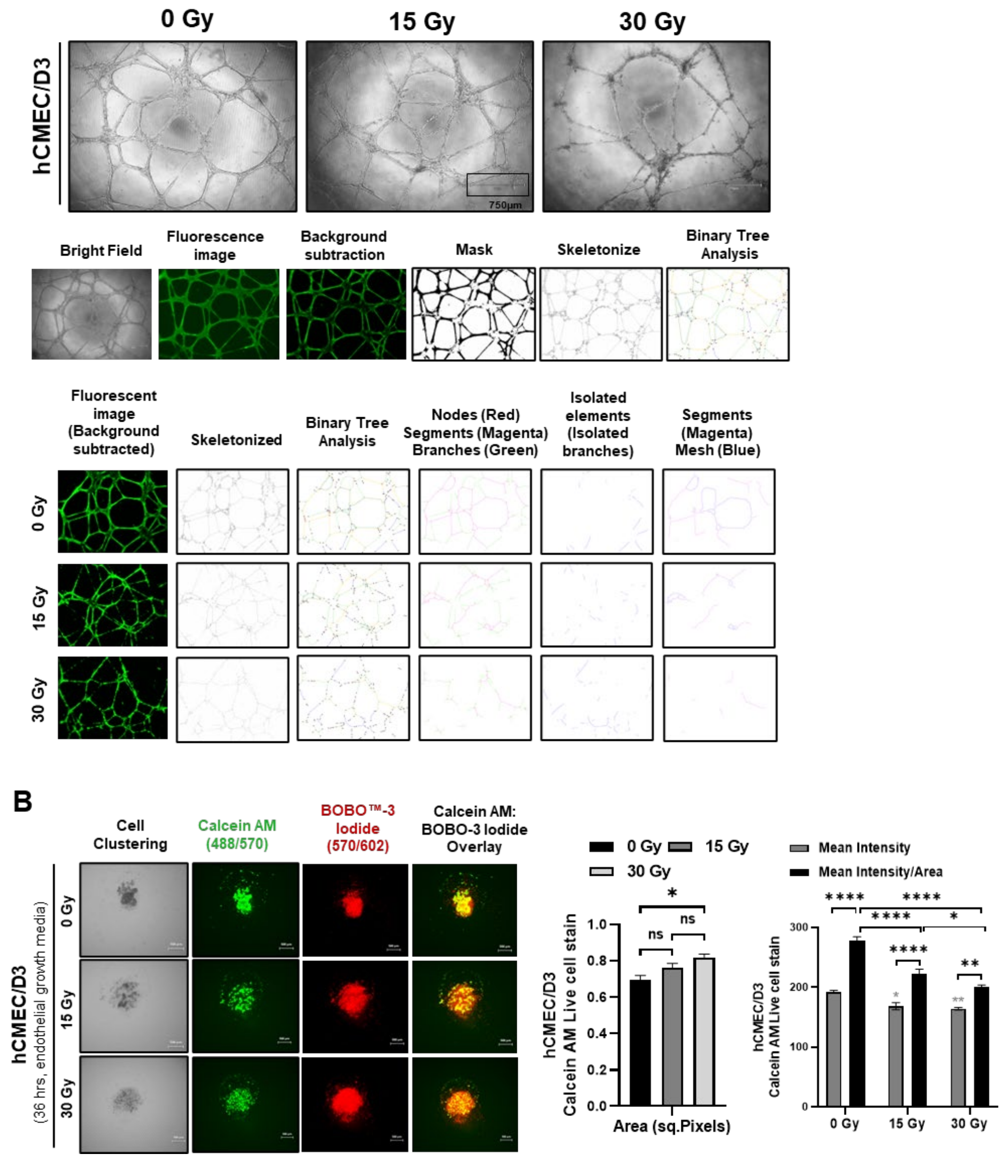

Supplementary Figure 3. A, Endothelial cell tube formation of hCMEC/D3 cells was assessed 24 hrs after IR at 15 and 30 Gy with stepwise methodology utilized to annotate various angiogenic parameters using ImageJ. B, Compact spheroid formation and enhanced spheroid disruption were assessed by staining with calcein AM and BOBO3-i 36 hrs after IR at 10, 15, and 30 Gy in hCMEC/D3 cells. The data are presented as the means ± SEMs (n=3/group); *p < 0.05, **p < 0.01, ***p <0.001, and ****p < 0.0001 according to one-way ANOVA or two-way ANOVA with Tukey's test.

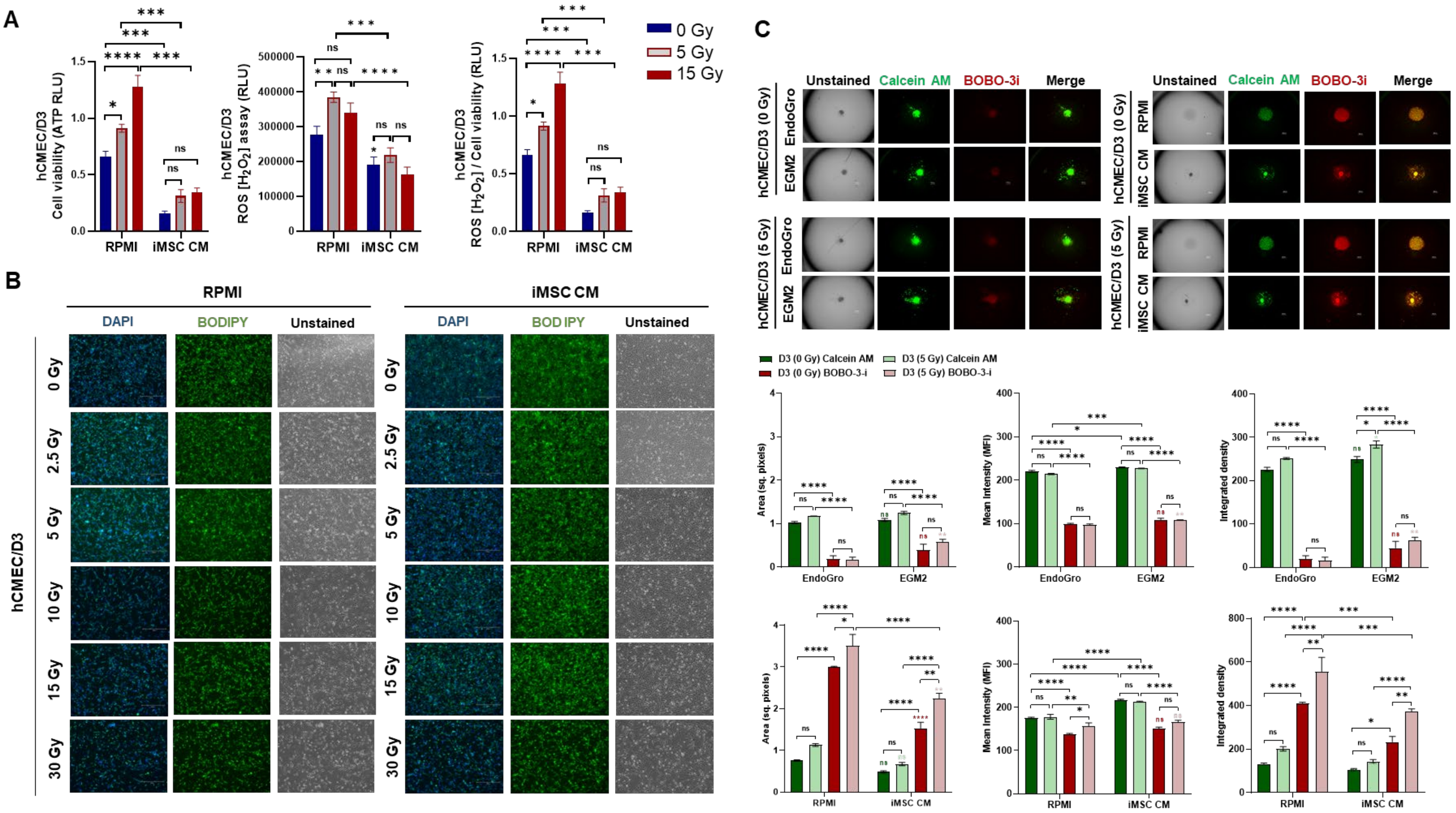

Supplementary Figure 4. A, Cell viability of hCMEC/D3 cells was assessed by measuring ATP production and ROS production 24 hrs after IR (0-15 Gy) with or without iMSC CM treatment. ROS production was normalized by cell viability. B, hCMEC/D3 cell adherence was assessed by staining for DAPI and BODIPY 24 hrs after IR (0-30 Gy) with or without iMSC CM treatment. C, hCMEC/D3 cells were irradiated at 5 Gy and cultured in U-bottom 96 well plate in complete endothelial growth media (EndoGro and EGM2), RPMI medium, and iMSC CM. Spheroid integrity was assessed using calcein AM and BOBO3-i (Live/Dead staining). The data are presented as the means ± SEMs (n=3/group); *p < 0.05, **p < 0.01, ***p <0.001, and ****p < 0.0001 according to one-way ANOVA or two-way ANOVA with Tukey's test.

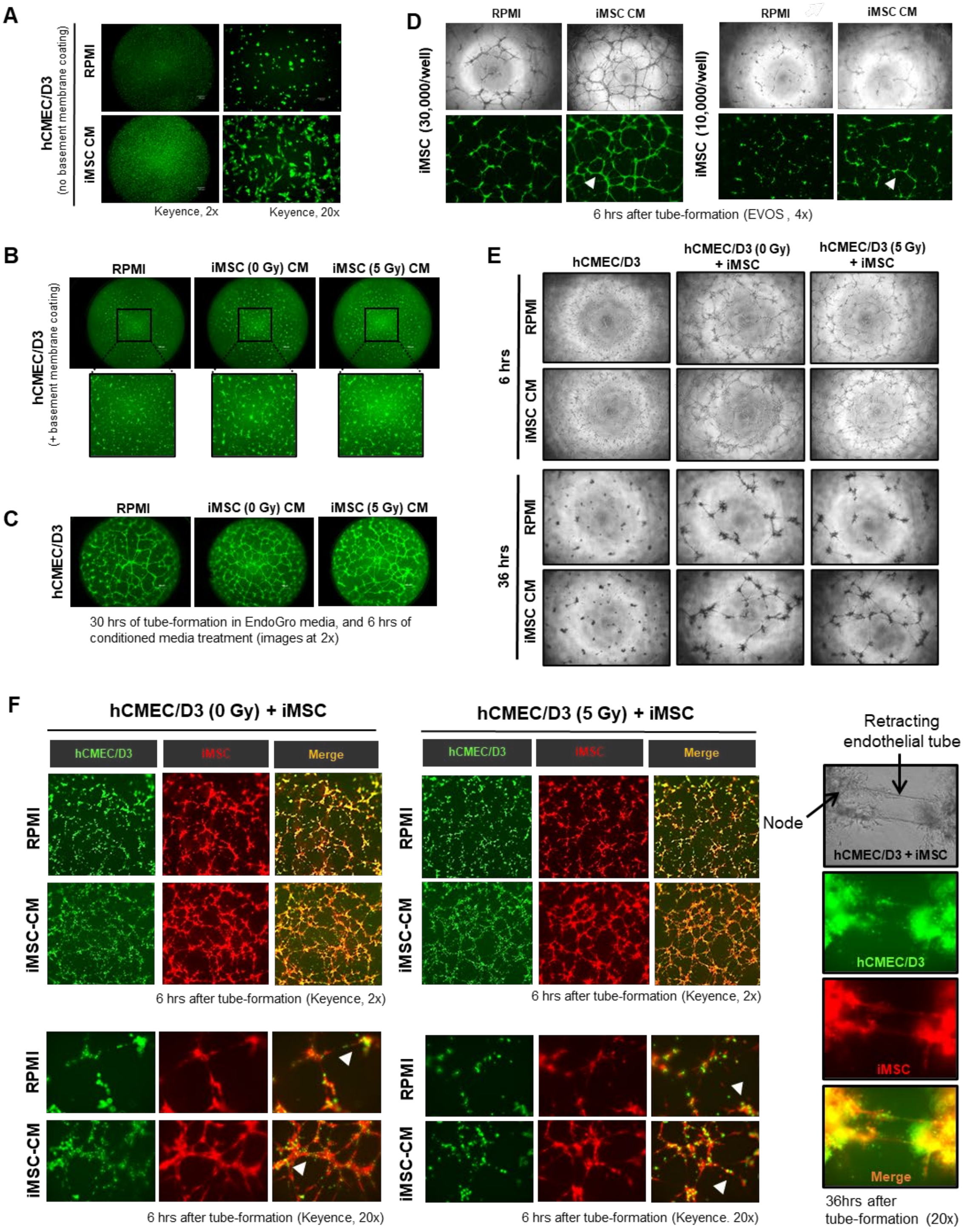

Supplementary Figure 5. A, Cell adhesion and spreading of hCMEC/D3 cells were cultured for 24 hrs on flat-bottom 96-well plates in presence of iMSC CM. B, CM was collected from iMSCs with or without IR at 5 Gy after conditioned for 5 days. Angiogenic sprouting and endothelial cell tube formation of hCMEC/D3 cells cultured on BME precoated 96-well plates were assessed by calcein AM staining 24 hrs after iMSC CM treatment. C, hCMEC/D3 cells were cultured on the precoated plates in complete endothelial growth media (EndoGro) for 30 hrs. The endothelial cell morphology was assessed after 6 hrs of treatment with CM collected from iMSC with or without IR administration. D, iMSCs (10,000 or 30,000 cells/well) were cultured on the precoated 96-well plates for 24 hrs. The morphology was assessed 6 hrs after iMSC CM treatment. E, hCMEC/D3 cells (30,000 cells/well) cultured on the precoated 96-well plates for 24 hrs and irradiated at 5 Gy. The morphology was assessed 6 and 36 hrs after the treatment with or without iMSC CM treatment. In addition, hCMEC/D3 cells (20,000 cells/well) were cultured on the precoated 96-well plates for 24 hrs and irradiated at 5 Gy. The morphology was assessed 6 and 36 hrs after co-culture with iMSCs (10,000 cells/well) with or without iMSC CM treatment. F, hCMEC/D3 cells and iMSCs were stained with calcein AM (green) and cell-tracker red, respectively. hCMEC/D3 cells (20,000 cells/well) and iMSCs (10,000 cells/well) were co-cultured on the precoated 96-well plates for 48 hrs in the presence or absence of iMSC CM. The morphology was assessed at 6 hrs and 36 hrs after iMSC CM treatment. All images are representative of at least two independent set of experiments.

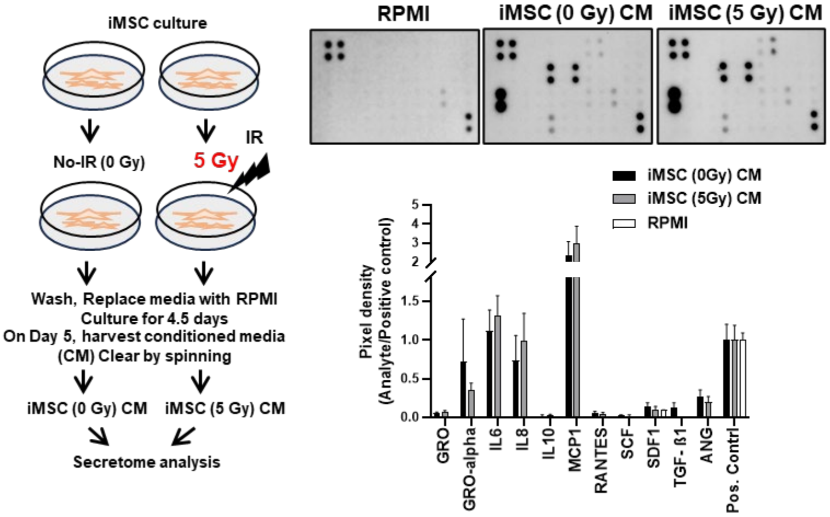

Supplementary Figure 6. CM was collected from iMSCs with or without IR at 5 Gy after conditioned for 5 days. Components of iMSC CM were analyzed by the cytokine antibody array (n=2/group). RPMI medium was used as a control. The data are presented as the means ± SEMs.

A

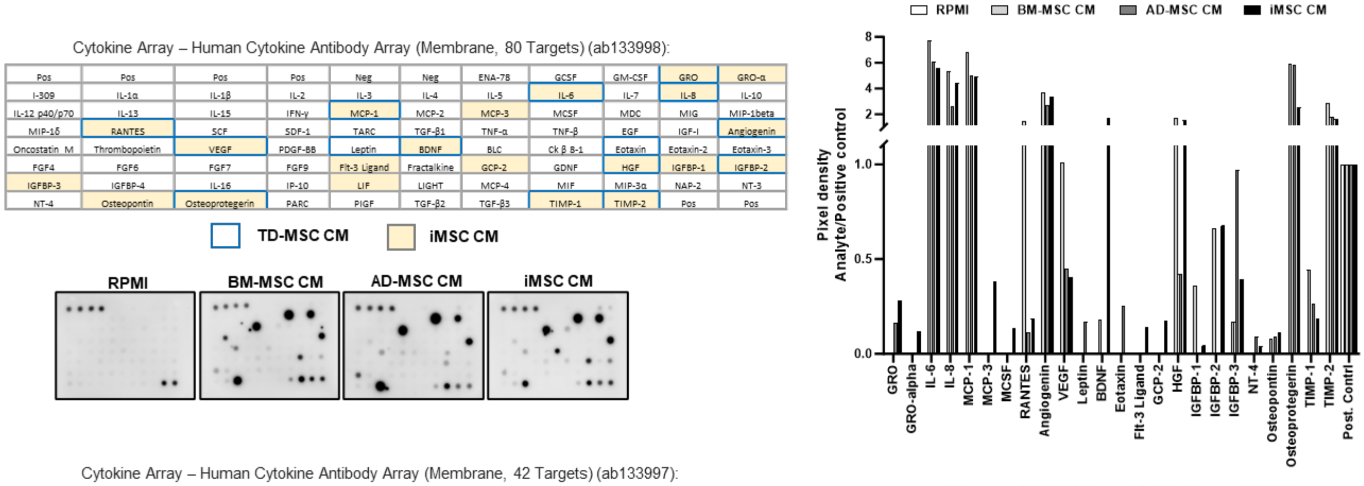

B

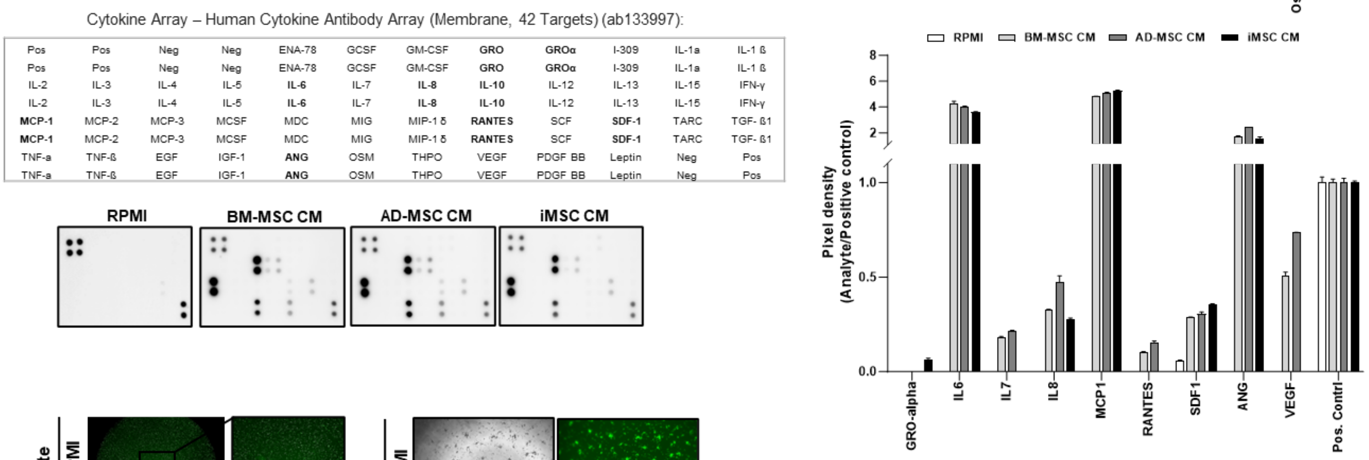

C

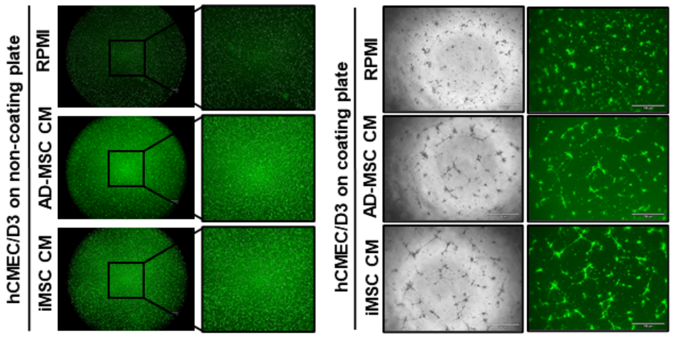

Supplementary Figure 7. A, CM was collected from iMSCs, BM-MSC, and AD-MSCs after conditioned for 5 days, and subjected to dot-blotting for human cytokine antibody array (80 targets/membrane). Scheme of targets spotted on the cytokine array is shown. B, CM was collected from iMSCs, AD-MSC, BM-MSCs and fibroblast (FB) after conditioned for 7 days. Components of CM from iMSC, AD-MSC, and BM-MSC were analyzed using human cytokine antibody array (42 targets/membrane). Scheme of targets spotted on the cytokine array is shown. C, hCMEC/D3 cells (30,000 cells/well) were cultured on 96-well plates with or without BME precoating in the presence of iMSC CM or AD-MSC CM for 24 hrs.

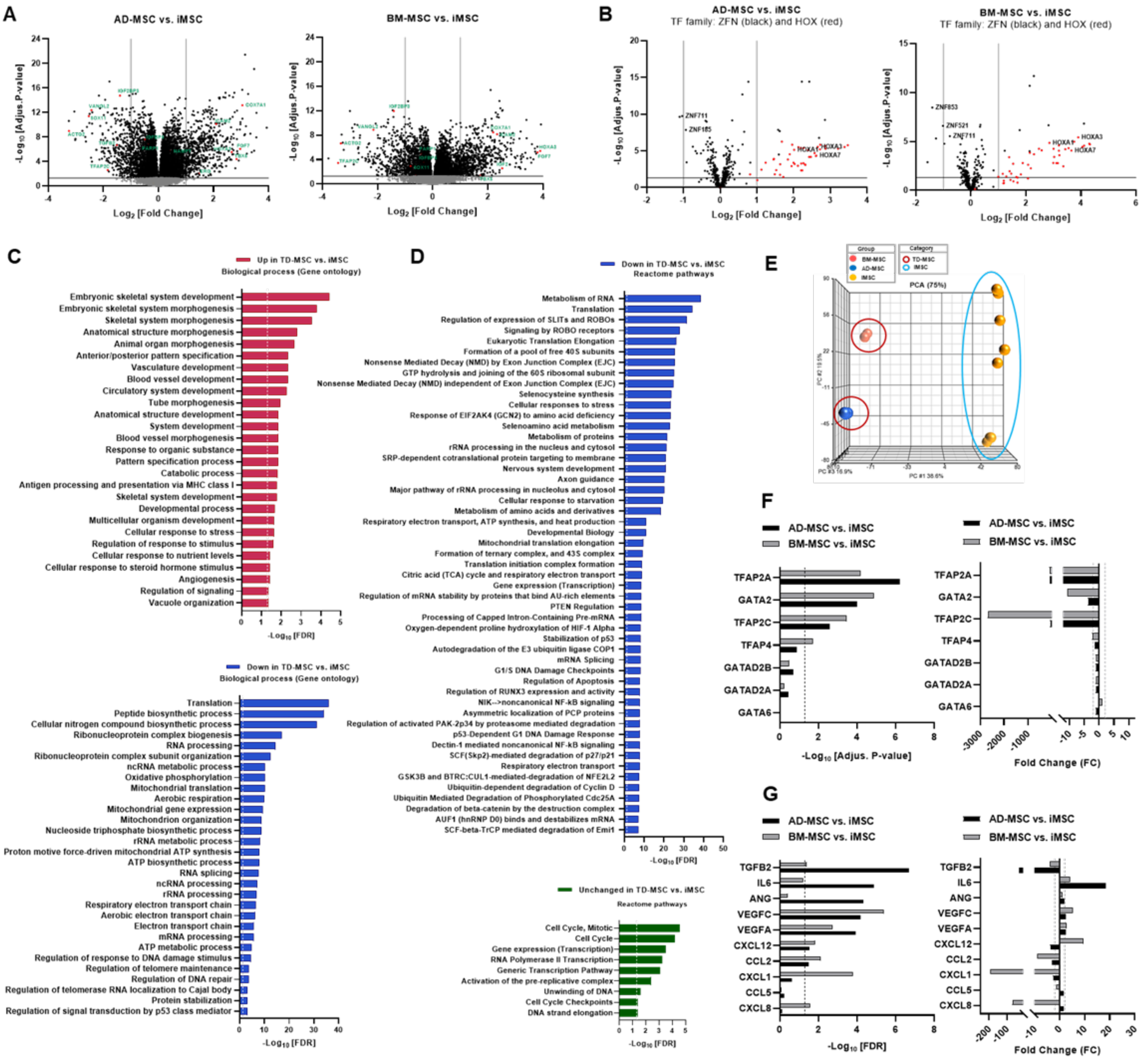

Supplementary Figure 8. RNA-seq analysis of iMSCs and tissue-derived MSCs. Transcriptome analysis was performed in iMSCs, AD-MSCs, and BM-MSCs using bulk RNA-seq. A, Volcano plots for genes differentially regulated in AD-MSCs and BM-MSCs compared to iMSCs are shown. B, Volcano plot for genes related to zinc finger nucleases (ZNF) and homeobox (HOX) family genes differentially regulated in AD-MSCs and BM-MSCs compared to iMSCs are shown. C, D, Functional annotation was performed for top 2000 genes upregulated or downregulated in tissue- derived MSCs (AD-MSCs and BM-MSCs) compared to iMSCs using STRING database. Top-30 biological processes (GO) upregulated or downregulated pathways (C) and top-50 downregulated or unchanged reactome pathways (D) are shown. E, Principal component analysis for three individual groups, AD-MSCs, BM-MSCs, and iMSCs is shown. F, Adjusted P-value and fold change for TFAP2 and GATA family genes differentially regulated in AD-MSCs and BM-MSCs compared to iMSCs are shown. G, FDR and fold change for genes encoding pro-angiogenic and immunomodulatory molecules differentially regulated in AD-MSCs and BM-MSCs compared to iMSCs are shown.

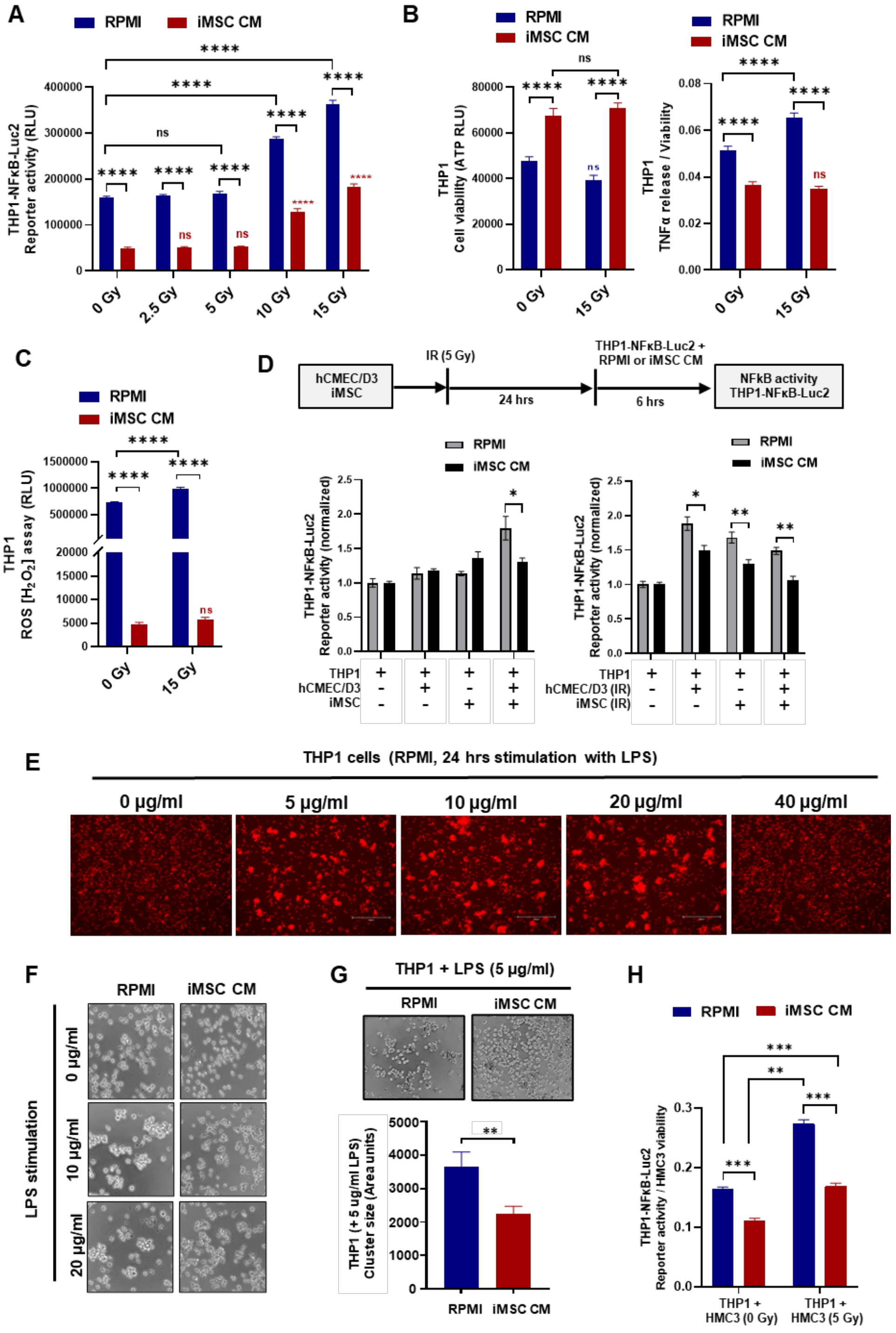

Supplementary Figure 9. A, NF-κB promoter activity in THP1-NF-κB-Luc2 cells was assessed 24 hrs after IR (0-15 Gy) with or without iMSC CM treatment (n=5/group). B, Cell viability of THP1 cells was assessed by measuring ATP production 24 hrs after IR at 15 Gy with or without iMSC CM treatment. TNFα levels in the THP1 culture medium were normalized by the cell viability (n=5/group). C, ROS production in THP1 cells was measured 24 hrs after IR at 15 Gy with or without iMSC CM treatment (n=5/group). D, hCMEC/D3 cells and/or iMSCs were irradiated at 5 Gy. After 24 hrs, they were co-cultured with THP1-NF-κB-Luc2 cells with or without iMSC CM treatment for 6 hrs, followed by NF-κB promoter activity measurements (n=5/group). E, THP1 cells were stained with cell tracker red after LPS stimulation (0-40 µg/ml) for 24 hrs. F, Clustering of THP1 cells was assessed after LPS stimulation (0-20 µg/ml) for 24 hrs with or without iMSC CM treatment. G, Clustering of THP1 cells was quantified after LPS stimulation (5 µg/ml) for 24 hrs in the presence of RPMI medium (n=62 regions) or iMSC CM (n=73 regions). H, HMC3 cells were irradiated at 5 Gy. After 48 hrs, the irradiated cells were co-cultured with THP1-NF-κB-Luc2 cells with or without iMSC CM treatment for 6 hrs, followed by NF-κB promoter activity measurements (n=5/group). RPMI medium was used as a control. The data are presented as the means ± SEMs; *$p < 0.05$, **$p < 0.01$, ***$p < 0.001$, and ****$p < 0.0001$ according to one-way ANOVA or two-way ANOVA with Tukey's test.

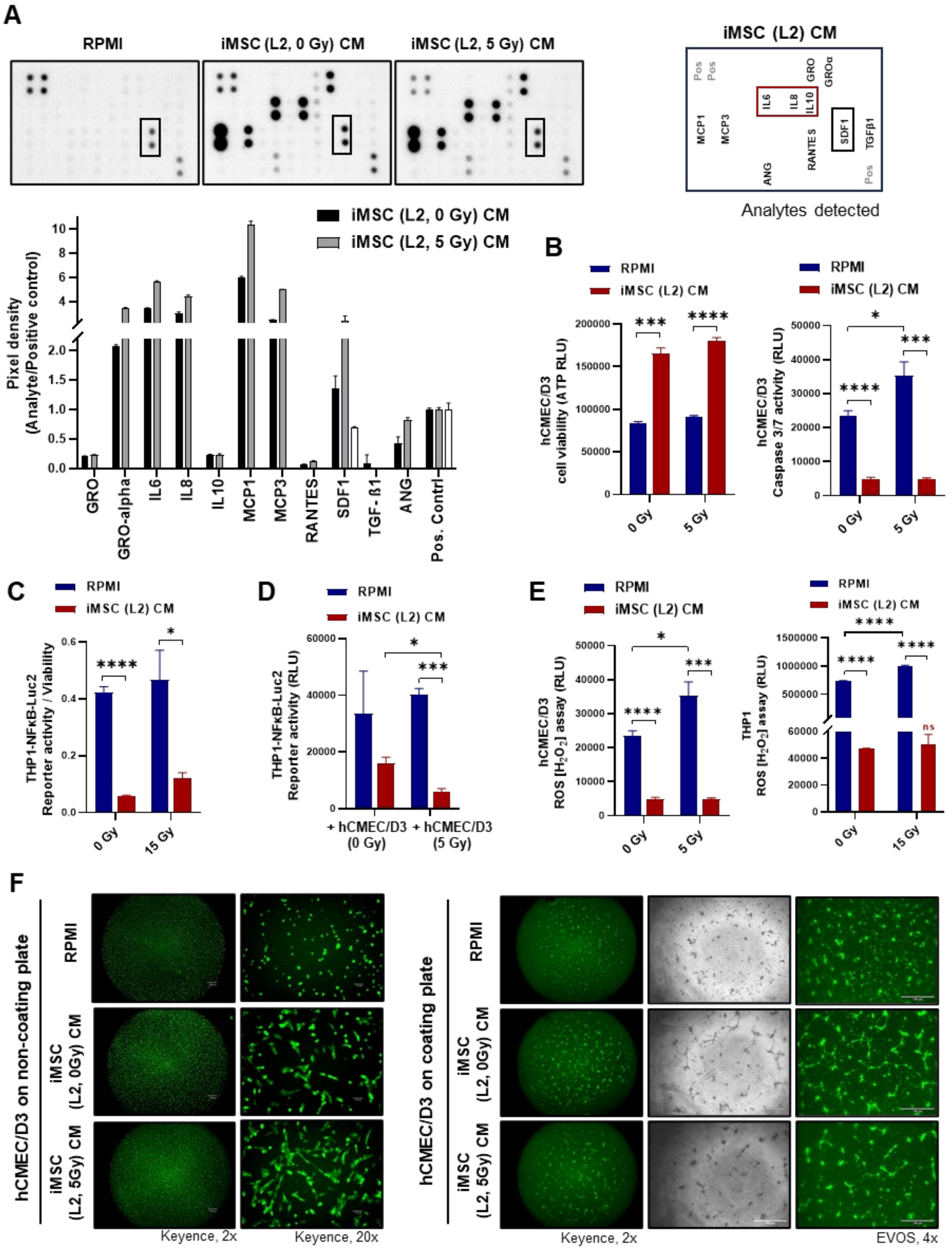

Supplementary Figure 10. To confirm the phenotypes of iMSCs, another independent iPSC line (MC0063) was differentiated into iMSCs (L2). A, CM was collected from iMSCs (L2) with or without IR at 5 Gy after conditioned for 5 days. Components of iMSC (L2) CM were analyzed by the cytokine antibody array (n=1/group). B, Cell viability and apoptosis of hCMEC/D3 cells were assessed by measuring ATP production and caspase 3/7 activity assay, respectively, 24 hrs after IR at 5 Gy with or without iMSC (L2) CM treatment. C, Cell viability of THP1 cells was assessed by measuring ATP production 24 hrs after IR at 15 Gy with or without iMSC (L2) CM treatment. TNFα levels in the THP1 culture medium were normalized by the cell viability. D, hCMEC/D3 cells were irradiated at 5 Gy. After 24 hrs, the cells were co-cultured with THP1-NF-κB-Luc2 cells with or without iMSC (L2) CM treatment for 6 hrs, followed by NF-κB promoter activity measurements. E, ROS production in hCMEC/D3 cells was measured 24 hrs after IR at 5 or 15 Gy with or without iMSC (L2) CM treatment. F, CM was collected from iMSCs (L2) with or without IR at 5 Gy after conditioned for 5 days. hCMEC/D3 cells (30,000 cells/well) were cultured on 96-well plates with or without BME precoating in the presence or absence of iMSC (L2) CM for 24 hrs. Angiogenic sprouting and endothelial cell tube formation were assessed by calcein AM staining. RPMI medium was used as a control. The data are presented as the means ± SEMs (n=5/group); *p < 0.05, **p < 0.01, ***p <0.001, and ****p < 0.0001 according to one-way ANOVA or two-way ANOVA with Tukey's test.

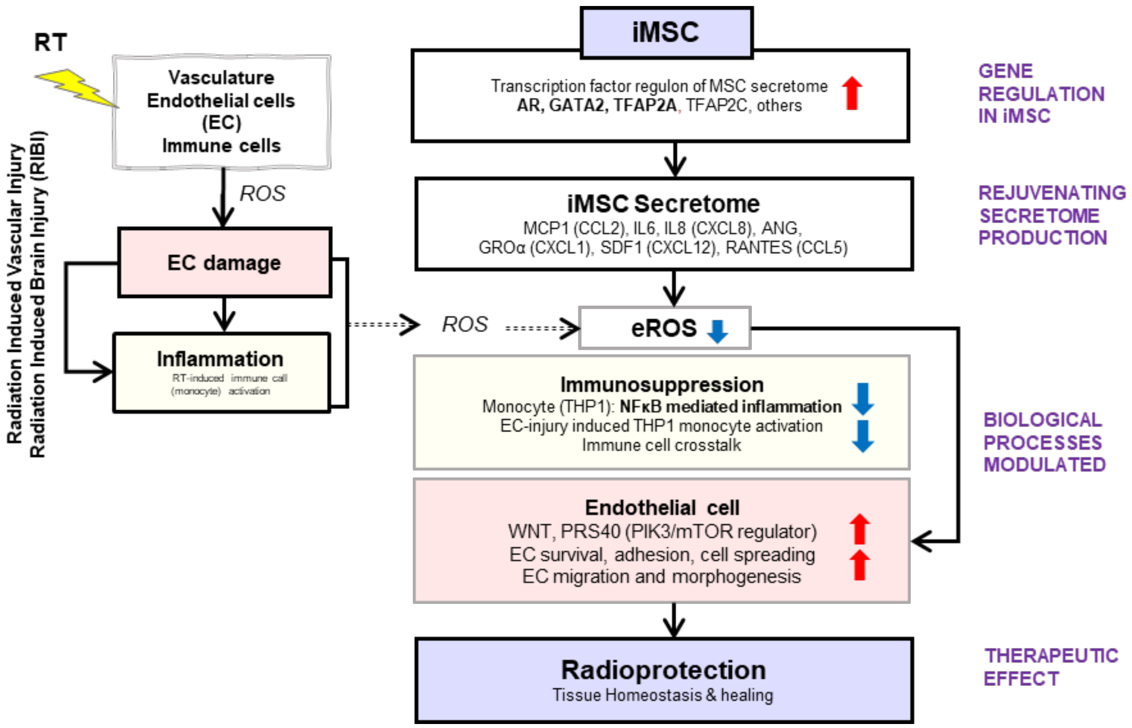

Supplementary Figure 11. Possible radioprotection mechanisms in iMSC secretome administration to treat RIBI.
